# Bi-Objective Modelling for Hazardous Materials Road–Rail Multimodal Routing Problem with Railway Schedule-Based Space–Time Constraints

**DOI:** 10.3390/ijerph13080762

**Published:** 2016-07-28

**Authors:** Yan Sun, Maoxiang Lang, Danzhu Wang

**Affiliations:** 1 School of Traffic and Transportation, Beijing Jiaotong University, Beijing 100044, China; sunyanbjtu@163.com; 2 Ministry of Education Key Laboratory for Urban Transportation Complex Systems Theory and Technology, Beijing Jiaotong University, Beijing 100044, China; 3 Transportation and Economics Research Institute, China Academy of Railway Sciences, Beijing 100081, China; wangdanzhu@rails.cn

**Keywords:** hazardous materials, multimodal routing, risk evaluation, bi-objective optimization, linear reformulation

## Abstract

The transportation of hazardous materials is always accompanied by considerable risk that will impact public and environment security. As an efficient and reliable transportation organization, a multimodal service should participate in the transportation of hazardous materials. In this study, we focus on transporting hazardous materials through the multimodal service network and explore the hazardous materials multimodal routing problem from the operational level of network planning. To formulate this problem more practicably, minimizing the total generalized costs of transporting the hazardous materials and the social risk along the planned routes are set as the optimization objectives. Meanwhile, the following formulation characteristics will be comprehensively modelled: (1) specific customer demands; (2) multiple hazardous material flows; (3) capacitated schedule-based rail service and uncapacitated time-flexible road service; and (4) environmental risk constraint. A bi-objective mixed integer nonlinear programming model is first built to formulate the routing problem that combines the formulation characteristics above. Then linear reformations are developed to linearize and improve the initial model so that it can be effectively solved by exact solution algorithms on standard mathematical programming software. By utilizing the normalized weighted sum method, we can generate the Pareto solutions to the bi-objective optimization problem for a specific case. Finally, a large-scale empirical case study from the Beijing–Tianjin–Hebei Region in China is presented to demonstrate the feasibility of the proposed methods in dealing with the practical problem. Various scenarios are also discussed in the case study.

## 1. Introduction

Transportation of hazardous materials has always been a highlighted problem in both the public security and transportation planning fields. Hazardous materials have five characteristics: corrosivity, toxicity, ignitability, reactivity, and infectivity [1]. Therefore, unlike regular goods or materials transportation, the transportation of hazardous materials often involves many risk factors [2]. Once accidents happen in the transportation of hazardous materials, severe consequences, such as loss of life, public damage (e.g., environment pollution, infrastructure unavailability, and economic losses) [3], and social instability, will emerge. In China, hazardous materials are plentiful. The freight volume of hazardous materials reaches more than 4 billion tons per year [4]. However, accidents happen frequently, e.g., transportation accidents involving chemicals account for 30%–40% of total accidents involving hazardous chemicals [4] and cause 3% of fatalities [5], which tremendously affects the normal operation of the social system. Also, the transportation of hazardous materials is difficult to avoid, and, according to statistics, 95% of hazardous materials in China need to be transported between different regions [4,6]. As a result, great importance has been attached to the effective management of the transportation of hazardous materials not only by the Chinese government but also by researchers in the field of transportation planning.

It has been widely recognized that the risk of the transportation of hazardous materials can be considerably reduced by advanced routing that minimizes accident probability and/or the expected consequences of an accident [7,8]. Considerable attention to this fact has been paid by many countries. Taking the United States as an example, the Hazardous Materials Transportation Uniform Act of 1993 emphasized that decision-making on routing is a way of reducing public risk from the transportation of hazardous materials [2]. Consequently, studies on the hazardous materials routing problem have been carried out in recent decades.

The majority (approx. 80% [4,6,9]) of the transportation of hazardous materials is served by road. Many publications have explored the relevant routing problem in the road network based on the well-known Vehicle Routing Problem. Some of them were from a tactical level (network design viewpoint), e.g., Verter and Kara [10], Erkut and Gzara [11], Kara and Verter [12], Erkut and Alp [13], Zhao et al. [1], and so on. Others were from an operational level and considered specific customer demands, e.g., Tarantilis and Kiranoudis [2], Androutsopoulos and Zografos [7], Boyer et al. [14], and so on. Moreover, many studies stressed the siting of disposal or treatment facilities for hazardous wastes when routing such materials, e.g., Boyer et al. [14], Alumur and Kara [15], Zhao and Verter [16], Shuai and Zhao [17], Helander and Melachrinoudis [18], Giannikos [19], Zografros and Samara [20], and so on. In this case, the routing problem of hazardous materials is extended to the location-routing problem of hazardous materials.

Actually in practice, for the transportation of hazardous materials, compared with road services, rail services have proved to achieve higher safety and lower risk [3,21]. Hence, they should take corresponding responsibilities and actively participate in the transportation of hazardous materials. Moreover, the recent U.S. commodity flow survey suggests that the transportation of hazardous materials often involves road–rail multimodal service, especially for long-haul transportation [16]. Additionally, many case studies have also indicated the necessity of multimodal mode in the transportation of hazardous materials [3]. However, so far, as mentioned above, almost all the current studies of the routing problem of hazardous materials dealt with the single mode, usually the road services.

Currently, Xie et al. [22] and Jiang et al. [23] independently explored the road–rail multimodal location-routing problem of hazardous materials from the viewpoint of multi-commodity flow. In their studies, the risk was evaluated in terms of the population exposure (the population within the potential impact zones along the hazardous materials routes). To deal with the cost–risk bi-objective optimization, different weights were distributed to the cost and risk objectives. Then the two weighted objectives were linearly combined to transform the bi-objective optimization into a single objective one. Nonlinear constraints in their models were linearized by inequalities, so that the location-routing problem could be effectively solved by any standard mathematical programming software. Finally, the weight-based solutions of the cases were generated to better support the decision-making. Moreover, in Xie et al.’s study [22], the risk was also controlled by the given risk thresholds, which means the risk at the nodes and on the arcs should not exceed determined values.

Both of these studies are from a tactical level (network design viewpoint). The specific customer demands are not fully considered and the schedules of the freight trains are not formulated. As for the hazardous materials road–rail multimodal routing problem, at the operational level, only two studies can be found, e.g., Verma and Verter [24] and Verma et al. [25]. However, neither of the studies formulate rail services as a kind of scheduled-based service. Besides, in their studies, the solution strategies are based on heuristic algorithms that are an approximate solution method. How to solve the problem exactly and how to test the performance of the heuristic algorithm are not indicated in their studies.

In this study, in order to further develop the road–rail multimodal routing problem of hazardous materials, we discuss it from the operational level of network planning, and comprehensively consider the following formulation characteristics.

(1) Customer demands, including origins, destinations, volumes, release times, and due dates.
(2) Multiple hazardous materials flows but single type of hazardous materials, i.e., multiple origin–destination pairs.
(3) Capacitated schedule-based rail services and uncapacitated time-flexible road services [26]. In particular, the restrictions of railway schedules on the routing decision are formulated as the railway schedule-based space–time constraints.
(4) Environmental risk constraint to lower and balance the environmental risk under a given threshold.
(5) Bi-objective optimization, including minimizing the total generalized costs of transporting the multiple hazardous materials and the social risk along the planned routes.

The remaining sections of this study are organized as follows. In Section 2, the specific transportation scenario for the hazardous materials and some background information are introduced and described, and the railway schedule-based space–time constraints are presented and explained. In Section 3, we evaluate and formulate the transportation risk of hazardous materials from the viewpoints of society and the environment. In Section 4, we build a bi-objective mixed integer nonlinear programming to model the road–rail multimodal routing problem of hazardous materials that comprehensively considers the above five characteristics. In Section 5, linear reformulations are developed to gain the equivalent linear programming of the initial model so that it can be effectively solved by exact solution algorithms on standard mathematical programming software. The normalized weighted sum method is also introduced in this section to address the bi-objective optimization. In Section 6, an empirical case study from the Beijing–Tianjin–Hebei Region in China is designed to demonstrate the feasibility of the proposed methods in dealing with the practical problem. Some discussions on various scenarios are also presented in this section. Finally, the conclusions of this study are drawn in Section 7.

## 2. Transportation Scenario Description

The road–rail multimodal service network is composed of three kinds of nodes, including origins, destinations, and hazardous materials stations. The hazardous materials stations are also the transshipment nodes, where transshipments between rail services and road services and between different rail services are conducted. We focus on combining the rail services and road services to generate the optimal routes for transporting the multiple hazardous materials flows. The two kinds of services adopt different operation modes in the transportation practice. The operation of a rail service is based on its prescribed schedule, while a road service is a kind of flexible service [26].

For a certain rail service, its schedule formulates its operation from both time and space viewpoints. These include its route, loading/unloading operation time windows at the goods yards, and classification/disassembly operation windows at the marshalling yards of the stations on the scheduled route. Arrival times and departure times at and from these stations are also included. The two kinds of operation time windows are intervals from operation start times to corresponding cutoff times. In addition, due to the limitations of the effective length of the rail tracks and of the locomotives’ tractions, rail services are a kind of capacitated service. On the contrary, the organization of the road service is relatively flexible and is not restricted in terms of time and space. It is also easy to assign enough road vehicles to carry the hazardous materials. Hence, we formulate road services as a kind of uncapacitated service. Additionally, in this study, we neglect the operation times of loading/unloading hazardous materials on/from the trains and road vehicles as well as the operation times of classifying/disassembling rail wagons carrying the hazardous materials into/from the trains. That is, the hazardous materials will complete the unloading operation once they arrive at a node by road service, or complete unloading/disassembly operation at the corresponding unloading/disassembly start time at the node when arriving by the rail service.

As described above, the prescribed schedules of rail services restrict their operations from the space–time viewpoint. Cases 1–3 describe the railway schedule-based space–time constraints, and should be comprehensively modelled in hazardous materials routing.

**Case 1.** When adopting a certain rail service to transport hazardous materials from the current node to the successor node, the arrival time of the hazardous materials at the current node should not be later than the operation (loading/classification) cutoff time of the rail service at the same node. If the arrival time is earlier than the operation (loading/classification) start time, it should wait until that time at the current node, and then get processed further. This case has two sub-cases as follows:

**Case 1.1.** If the hazardous materials arrive at the current node by road service, they should be loaded on the rail service at the goods yard current node. Consequently, in Case 1, the operation start time and operation cutoff time separately refer to the loading start time and loading cutoff time of the rail service. In this case, the selection of a rail service is constrained by its loading operation time window at the current node, and the inventory costs and loading/unloading costs will be charged.

**Case 1.2.** If the hazardous materials arrive at the current node by another rail service, carried by rail wagons, they will get classified into the rail service at the marshalling yards of the current node, and there is no loading operation. Consequently, in Case 1, the operation start time and operation cutoff time separately refer to the classification start time and classification cutoff time of the rail service. In this case, the selection of a rail service is constrained by its classification operation time window at the current node. Contrary to Case 1.1, because there are no loading/unloading operations and inventory services in this case, corresponding costs do not exist.

**Case 2.** The hazardous materials will not depart from the current node once the loading/classification operation is completed. They will wait until the scheduled departure time of the rail service at the same node, and then depart from the current node along with the train.

**Case 3.** The hazardous materials will arrive at the successor node covered on the scheduled route along with the rail service at its scheduled arrival time at the same node. After arriving at the node, they cannot get unloaded/disassembled immediately and should wait until the unloading/disassembled operation start time of the rail service at the same node.

Contrary to the rail service, the organization of the road service is time-flexible. Transshipment is unnecessary if the hazardous materials both arrive at and then depart from the node by road services. A road service route can be entirely or partly covered in the hazardous materials multimodal routes. For the convenience of modelling, we can divide the road service routes into several segments, e.g., a road service route (1, 2, 3, 4) is divided into (1, 2), (1, 3), (1, 4), (2, 3), (2, 4), and (3, 4). The adjacent segments should not be covered in a hazardous materials route at the same time to avoid extra loading/unloading costs and transportation delay. Furthermore, there are no such constraints (Case 1 to Case 3) above when utilizing the road service to transport hazardous materials from the current node to the successor node. Consequently, there exist the following Cases 4 and 5 for road service in the multimodal service network.

**Case 4.** The hazardous materials can immediately be loaded onto the road vehicles once they arrive at the current node. Once the loading operation is finished, the hazardous materials can immediately depart from the current node.

**Case 5.** The hazardous materials can immediately be unloaded from the road vehicles once they arrive at the successor node.

## 3. Risk Evaluation and Modelling

In order to manage and control the potential risk, risk evaluation on the planned route for the transportation of hazardous materials is quite important and necessary in decision-making. People and the environment are the key factors and indexes to evaluate the risk.

### 3.1. Social Risk Evaluation

The purpose of social risk evaluation is to determine how many people along the transportation routes of hazardous materials will be potentially impacted and how much the degree of potential risk will be. The degree can be reflected by the volume of the hazardous materials: the larger the volume, the higher the risk degree will be. We can multiply the population exposure and its corresponding volume of the hazardous materials in order comprehensively to express the two aspects of the social risk quantitatively, i.e.,

Social risk = population exposure × volume of the hazardous materials

The population exposure can be calculated from the population density and exposure area (area of the potential dispersion zone of the hazardous materials once an accident occurs). The exposure area is determined by the dispersion distance threshold in the accidental release of hazardous materials. The dispersion distance is related to the spatial distribution of the toxic concentration level. In addition, such spatial distribution is influenced by various factors, including the types of the hazardous materials, atmospheric and geographical conditions of the accident, and so on [27]. Specifically, in this study, we consider that hazardous materials such as chlorine and methane become airborne in the accidental release.

There are many models for estimating such spatial distribution, among which the Gaussian plume model is the one commonly used [18]. Also, many standards formulated by the U.S. Environmental Protection Agency refer to this model [28,29]. So, in this study, we adopt the Gaussian plume model to estimate the spatial distribution of the toxic concentration level. The Gaussian plume model comprehensively considers the distance to the release source, the release rate, and the wind speed and direction in order to evaluate the toxic concentration level of the hazardous materials in the accidental release [24]. It assumes that the release rate of the hazardous materials and the surrounding atmospheric conditions remain constant during the dispersion process. When the release source and impact zone are at zero elevation, the concentration C(x) (unit: mg/m^3^) at downwind distance x to the release source is formulated by Equation (1) [17]:

(1) $$C\left(x\right)=\frac{Q}{\pi \cdot u\cdot \sigma_{y}\cdot \sigma_{z}}$$

In Equation (1), Q is the source release rate of the hazardous materials (unit: mg/s); u is the average downwind speed (unit: m/s); $\sigma_{y}$ and $\sigma_{z}$ are separately horizontal and vertical dispersion coefficients, and are determined by the atmospheric stability category and downwind distance x. There exist $\sigma_{y}=a\cdot x^{b}$ and $\sigma_{z}=c\cdot x^{d}$, where a, b, c, and d are the dispersion parameters. In China, their values are formulated by the Technical Methods for Making Local Emission standards for Air Pollutants (GB/T 3840-91) according to the atmospheric stability category and downwind distance. Q is determined by various factors, including the type of the hazardous materials, the size of the damaged container, the pressure and temperature inside the container, and so on. Let VOL and T denote the maximal volume that a container can carry and the release duration, respectively. The estimation of Q can be simplified by Equation (2) [17]:

(2) $$Q=\frac{VOL}{T}$$

For each hazardous material, the concentration threshold $\bar{C}$ that is immediately dangerous to life and health (IDLH concentration threshold) is known. By using this threshold value and modifying Equation (1), we can get the impact distance threshold $\bar{x}$ from Equation (3):

(3) $$\bar{x}=\sqrt[{c+d}]{\frac{Q}{\pi \cdot u\cdot a\cdot b\cdot \bar{C}}}$$

In this study, we consider that the potential dispersion direction of the hazardous materials in the accidental release is 360°. When evaluating the population exposure, we should consider the worst cases at the nodes and on the arcs. For node i, its population exposure is given by Equation (4), where $\rho_{i}$ is the population density around node i:

(4) $$POP_{i}=\rho_{i}\cdot {\left(\sqrt[{c+d}]{\frac{Q}{\pi \cdot u\cdot a\cdot b\cdot \bar{C}}}\right)}^{2}$$

For arc (i, j), when using service s to transport the hazardous materials, its population exposure is given by Equation 5, where $\rho_{ijs}$ is the population density along path (i, j, s) and $d_{ijs}$ is the transportation distance of service s on arc (i, j):

(5) $$POP_{ijs}=\rho_{ijs}\cdot d_{ijs}\cdot 2\cdot \sqrt[{c+d}]{\frac{Q}{\pi \cdot u\cdot a\cdot b\cdot \bar{C}}}$$

### 3.2. Environmental Risk Evaluation

Besides people, the environment will also be potentially impacted by the transportation of hazardous materials. For airborne hazardous materials, once accidental release occurs, surrounding ambient air and some sensitive zones (e.g., nature reserves, forests, rivers, lakes, and farmland) will be polluted. If the release volume of the hazardous materials in the ambient air exceeds the environmental capacity, the environment will be permanently damaged and so is difficult to clean up. Therefore, the environmental capacity should be considered in the hazardous materials road–rail multimodal routing problem. When evaluating the environmental risk, we first calculate the environmental capacities of the nodes and of the arcs.

We simplify by assuming that the potential impact spaces at nodes and at arcs are as in the box model, i.e., the potential impact space at a node is hemispherical while that at an arc is semi-cylindrical. Under this assumption, we can easily estimate the environmental capacity as follows. For node i, environmental capacity is given by Equation (6):

(6) $$CAP_{i}=C^{*}\cdot \frac{1}{2}\cdot \frac{4}{3}\cdot \pi \cdot R^{*3}=\frac{2}{3}\cdot \pi \cdot C^{*}\cdot R^{*3}$$

For arc (i, j), when using service s to transport the hazardous materials, its environmental capacity is given by Equation (7):

(7) $$CAP_{ijs}=\frac{1}{2}\cdot C^{*}\cdot d_{ijs}\cdot \pi \cdot R^{*2}$$

where $C^{*}$ is the allowed emission concentration threshold. It is formulated by the Integrated Emission Standard of Air Pollutants (GB 16297-1996). $R^{*}$ is the potential impact radius of the hazardous materials when accidental release occurs.

After determining the environmental capacity, we can then evaluate the environmental risk according to the definition proposed by Zhao in her doctoral dissertation [30]. For node i, environment risk is given by Equation (8), where $q_{i}$ is the cumulative hazardous materials volume that node i takes:

(8) $$ER_{i}=\frac{q_{i}}{CAP_{i}}$$

For arc (i, j), when using service s to transport the hazardous materials, environmental risk is given by Equation (9), where $q_{ijs}$ is cumulative hazardous materials volume that path (i, j, s) takes:

(9) $$ER_{ijs}=\frac{q_{ijs}}{CAP_{ijs}}$$

The values of $ER_{i}$ and of $ER_{ijs}$ reflect the aggregation degrees of the risk at a certain node and on a certain path, respectively, and can also reflect the environmental risk distribution in the multimodal service network.

## 4. Mathematical Model

### 4.1. Notations

In this study, G=(N, A, S) represents a road–rail multimodal service network, where N, A, and S are the node set, the directed arc set, and transportation service set in the network, respectively. In addition, for hazardous materials flow k (k∈K, where K is the flow set), its transportation demands, including origin $o_{k}$, destination $d_{k}$, release time at origin $t_{\text{release}}^{k}$, due date $T_{k}$, and the volume $q_{k}$ (unit: ton), are all determined and known in the location-routing problem. The remaining symbols in the model and their representations are listed as follows. Some of the following symbols are similar to our previous study [26].

- **Indices**

h,i, j− index of the nodes, and h,i, j∈N.

s,r− index of the transportation services, and s,r∈S.

(i, j)− directed arc from node i to node j, and (i, j)∈A.

(i, j,s)− path from node i to node j served by service s.

- **Sets**

$S_{ij}-$ set of transportation services on arc (i, j), $S_{ij}\subseteq S$ and $S_{ij}=\Gamma_{ij}\cup^{\text{​}}\Omega_{ij}$, where $\Gamma_{ij}$ and $\Omega_{ij}$ are separately the rail service set and road service set on arc (i, j).

$\delta^{-}\left(i\right)-$ predecessor node set to terminal i, and $\delta^{-}\left(i\right)\subseteq N$.

$\delta^{+}\left(i\right)-$ successor node set to terminal i, and $\delta^{+}\left(i\right)\subseteq N$.

- **Parameters**

$\left[e_{i}^{s},f_{i}^{s}\;\right]–$ classification/disassembly operation time window of rail service s at node i, where $e_{i}^{s}$ is the operation start time and $f_{i}^{s}$ is the operation cutoff time.

$\left[l_{i}^{s},u_{i}^{s}\;\right]-$loading/unloading operation time window of rail service s at node i, where $l_{i}^{s}$ is the operation start time and $u_{i}^{s}$ is the operation cutoff time, and $i\in N_{2}$.

$SA_{i}^{s}-$ scheduled arrival time of rail service s at node i.

$SD_{i}^{s}-$ scheduled departure time of rail service s from node i.

$Q_{ijs}-$ capacity of rail service s on arc (i, j), unit: ton.

$d_{ijs}-$ transportation distance of path (i, j, s), unit: km.

$t_{ijs}-$ transportation time of service s on arc (i, j, ), unit: h.

$POP_{ijs}-$ population exposure along path (i, j, s).

$POP_{i}-$ population exposure around node i.

$CAP_{ijs}-$ environmental capacity of path (i, j, s), unit: ton.

$CAP_{i}-$ environmental capacity of node i, unit: ton.

$ER_{\text{max}}-$ environmental risk threshold for the multimodal service network.

$c_{ijs}-$ unit costs of transporting hazardous materials flow on arc (i, j) by service s, unit: ¥/ton.

$c_{s}-$ unit costs of loading/unloading operation of service s at the node, unit: ¥/ton. Specifically: $c_{\text{rail}}-$ unit costs of loading/unloading operation of rail service at nodes, unit: ¥/ton.

τ− inventory period free of charge, unit: h.

$c_{\text{store}}-$ unit inventory costs of rail service at node, unit: ¥/ton-h.

M− a significant large positive number.

- **Decision Variables**

$W_{i}^{k}-$ 0–1 decision variable. If node i is included in the route for hazardous materials flow k, $W_{i}^{k}$= 1; otherwise $W_{i}^{k}$ = 0.

$X_{ijs}^{k}-$ 0–1 decision variable. If hazardous materials flow k is transported along path (i, j,s), $X_{ijs}^{k}$ = 1; otherwise $X_{ijs}^{k}$ = 0.

$Y_{i}^{k}-$ arrival time of hazardous materials flow k at node i.

$Z_{ijs}^{k}-$ charged inventory time of hazardous materials flow k at node i before transported by rail service s on arc (i, j), unit: h.

### 4.2. A Node–Arc-Based Model

Using the symbols above, the specific routing problem is first formulated as a bi-objective mixed integer nonlinear programming model in Equations/Components (10)–(31).

- **Objective 1**
  (10) $$\text{minimize }\underset{k\in K}{\sum }\underset{\left(i,j\right)\in A}{\sum }\underset{s\in S_{ij}}{\sum }c_{ijs}\cdot q_{k}\cdot X_{ijs}^{k}$$
  (11) $$\begin{array}{c} +\underset{k\in K}{\sum }\underset{i\in N}{\sum }\left(\underset{j\in \delta^{+}\left(i\right)}{\sum }\underset{s\in S_{ij}}{\sum }c_{s}\cdot q_{k}\cdot X_{ijs}^{k}+\underset{h\in \delta^{-}\left(i\right)}{\sum }\underset{r\in S_{hi}}{\sum }c_{r}\cdot q_{k}\cdot X_{hir}^{k}\right)\\ -\underset{k\in K}{\sum }\underset{i\in N\backslash \left\{o_{k},\;d_{k}\right\}}{\sum }\left(2\cdot c_{\text{rail}}\cdot q_{k}\cdot \text{max}\left\{\underset{h\in \delta^{-}\left(i\right)}{\sum }\underset{r\in \Gamma_{hi}}{\sum }X_{hir}^{k}+\underset{j\in \delta^{+}\left(i\right)}{\sum }\underset{s\in \Gamma_{ij}}{\sum }X_{ijs}^{k}-1,\;0\right\}\right) \end{array}$$
  (12) $$+\underset{k\in K}{\sum }\underset{\left(i,j\right)\in A}{\sum }\underset{s\in \Gamma_{ij}}{\sum }\left(c_{\text{store}}\cdot q_{k}\cdot Z_{ijs}^{k}\cdot \underset{h\in \delta^{-}\left(i\right)}{\sum }\underset{r\in \Omega_{ij}}{\sum }X_{hir}^{k}\right)$$

Objective 1 is to minimize the total generalized costs of routing the multiple hazardous material flows. Objective 1 consists of three components including the transportation costs (Component (10)), loading/unloading operation costs (Component (11)), and inventory costs (Component (12)) at the nodes. Objective 1 reflects the private goal of transportation demanders to pay the least capital to realize their transportation demands. Note that loading/unloading costs are only created (1) at the goods yards of the hazardous materials stations, where there are transshipments between road services and rail services; and (2) at the origins and destinations of the flows of hazardous materials. Therefore, the extra loading and unloading costs calculated in the transshipments between different rail services should be deducted. However, the inventory costs are only created in the first case. Consequently, we formulate the total loading/unloading costs and inventory costs as Components (11) and (12), respectively.

- **Objective 2**
  (13) $$\text{minimize }\underset{k\in K}{\sum }\underset{i\in N}{\sum }W_{i}^{k}\cdot POP_{i}\cdot q_{k}$$
  (14) $$+\underset{k\in K}{\sum }\underset{\left(i,j\right)\in A}{\sum }\underset{s\in S_{ij}}{\sum }X_{ijs}^{k}\cdot POP_{ijs}\cdot q_{k}$$

Objective 2 is to minimize the cumulative social risk along the planned routes for the transportation of hazardous materials. Objective 2 consists of two components, including the cumulative social risk at the nodes (Component (13)) and the cumulative social risk on the paths (Component (14)). Objective 2 reflects the public goal of lowering the potential impacted population and the potential risk degree in the multimodal service network.

- **Subject to**
  (15) $$\underset{j\in \delta^{+}\left(i\right)}{\sum }\underset{s\in S_{ij}}{\sum }X_{ijs}^{k}-\underset{h\in \delta^{-}\left(i\right)}{\sum }\underset{r\in S_{hi}}{\sum }X_{hir}^{k}=\{\begin{array}{cc} 1, & i=o_{k}\\ 0, & \forall i\in N\backslash \left\{o_{k},\;d_{k}\;\right\}\\ -1, & i=d_{k} \end{array}\;\forall k\in K,\;\forall i\in N$$
  (16) $$\underset{s\in S_{ij}}{\sum }X_{ijs}^{k}\leq 1\;\forall k\in K,\;\forall \left(i,\;j\right)\in A$$

Equation (15) is the general flow conservation constraint. The combination of Equations (15) and (16) ensures that each hazardous materials flow is non-bifurcated [31].

(17) $$\underset{h\in \delta^{-}\left(i\right)}{\sum }\underset{r\in \Omega_{hi}}{\sum }X_{hir}^{k}+\underset{j\in \delta^{+}\left(i\right)}{\sum }\underset{s\in \Omega_{ij}}{\sum }X_{ijs}^{k}\leq 1\;\forall k\in K,\;\forall i\in N\backslash \left\{o_{k},\;d_{k}\;\right\}$$

Equation (17) ensures that after dividing the road services route into several segments according to the method in Section 3.1, transshipments between road services are extra and unnecessary and should be avoided in the routing [26].

(18) $$W_{i}^{k}=\{\begin{array}{c} \begin{array}{cc} \sum_{j\in \delta^{+}\left(i\right)}\sum_{s\in S_{ij}}X_{ijs}^{k}, & \forall i\in N\backslash \left\{o_{k},\;d_{k}\;\right\} \end{array}\\ \begin{array}{cc} 1, & i\in \left\{o_{k},\;d_{k}\;\right\} \end{array} \end{array}\;\forall k\in K,\;\forall i\in N$$

Equation (18) ensures the compatibility requirements between decision variables $W_{i}^{k}$ and $X_{ijs}^{k}$. Because the origins and destinations are always included in the routes, for $i\in \left\{o_{k},\;d_{k}\;\right\}$ and ∀k∈K, there exists $W_{i}^{k}$ = 1. For the other nodes, if and only if a path linked with node i is selected for hazardous materials flow k, i.e., $\sum_{j\delta^{+}\left(i\right)}\sum_{s\in S_{ij}}X_{ijs}^{k}$ = 1, there exists $W_{i}^{k}$ = 1, otherwise $W_{i}^{k}$ = 0. Consequently, we formulate Equation (18) as above.

(19) $$\underset{i\in N}{\sum }\underset{k\in K}{\sum }\frac{W_{i}^{k}\cdot q_{k}}{CAP_{i}}+\underset{k\in K}{\sum }\underset{\left(i,j\right)\in A}{\sum }\underset{s\in S_{ij}}{\sum }\frac{X_{ijs}^{k}\cdot q_{k}}{CAP_{ijs}}\leq ER_{\text{max}}$$

Equation (19) is the environmental risk constraint. It ensures that the total environmental risk in the multimodal service network does not exceed a given threshold, so that the environmental risk distribution in the multimodal service network will be balanced and not excessively aggregated.

(20) $$\underset{k\in K}{\sum }q_{k}\cdot X_{ijs}^{k}\leq Q_{ijs}\;\forall \left(i,\;j\right)\in A,\;\forall s\in \Gamma_{ij}$$

Equation (20) is the rail service capacity constraint. It ensures that the loaded and classified volume of the hazardous materials should not exceed its available carrying capacity.

(21) $$\begin{array}{c} Y_{i}^{k}\leq u_{i}^{s}\cdot X_{ijs}^{k}\cdot \underset{h\in \delta^{-}\left(i\right)}{\sum }\underset{s\in \Omega_{ij}}{\sum }X_{hir}^{k}+f_{i}^{s}\cdot X_{ijs}^{k}\cdot \underset{h\in \delta^{-}\left(i\right)}{\sum }\underset{s\in \Gamma_{ij}}{\sum }X_{hir}^{k}\\ +M\cdot \left(1-X_{ijs}^{k}\right) \end{array}\;\forall \left(i,\;j\right)\in A,\;\forall s\in \Gamma_{ij}$$

Equation (21) is the operation time window constraint of the rail service that is adopted to move the hazardous materials from the current node to the successor node. This constraint contains three independent cases:

(i) If $X_{ijs}^{k}\;$= 0, $Y_{i}^{k}\leq M$ (which is always satisfied), which means the arrival time of the hazardous materials at the node is not restricted by the unselected rail services.
(ii) If $X_{ijs}^{k}\;$= 1 and $\sum_{h\in \delta^{-}\left(i\right)}\sum_{s\in \Omega_{ij}}X_{hir}^{k}\;$= 1, according to Equation (16), $\sum_{h\in \delta^{-}\left(i\right)}\sum_{s\in \Gamma_{ij}}X_{hir\;}^{k}$= 0, i.e., the hazardous materials k arrive at node i by road service, then according to Equation (21), $Y_{i}^{k}\leq u_{i}^{s}$, which matches the description of Case 1.1 in Section 2.
(iii) If $X_{ijs}^{k}\;$= 1 and $\sum_{h\in \delta^{-}\left(i\right)}\sum_{s\in \Gamma_{ij}}X_{hir}^{k}\;$= 1, just contrary to Equation (2), there is $Y_{i}^{k}\leq f_{i}^{s}$, which matches the description of Case 1.2 in Section 2.

(22) $$Y_{o_{k}}^{k}=t_{\text{release}}^{k}\;\forall k\in K$$

We assume that the arrival times of the flows of hazardous materials at their origins are their release times. Therefore, we have Equation (22) as above.

(23) $$\left(Y_{i}^{k}+t_{ijs}-Y_{j}^{k}\right)\cdot X_{ijs}^{k}=0\;\forall k\in K,\;\forall \left(i,j\right)\in A,\;\forall s\in \Omega_{ij}$$

(24) $$\left(l_{i}^{s}\cdot \underset{j\in \delta^{+}\left(i\right)}{\sum }\underset{s\in \Omega_{ij}}{\sum }X_{ijs}^{k}+e_{i}^{s}\cdot \underset{j\in \delta^{+}\left(i\right)}{\sum }\underset{s\in \Gamma_{ij}}{\sum }X_{ijs}^{k}-Y_{i}^{k}\right)\cdot X_{hir}^{k}=0\;\forall k\in K,\;\forall \left(h,i\right)\in A,\;\forall r\in \Gamma_{hi}$$

Equations (23) and (24) ensure the compatibility requirements between decision variables $X_{ijs}^{k}$ and $Y_{i}^{k}$. They calculate the arrival times of the flow of each hazardous material at the selected nodes. Especially for Equation (24), when adopting rail service r to transport hazardous materials k from predecessor node h to current node i ($X_{hir}^{k}\;$= 1):

(i) If the following transportation is by road service ($\sum_{j\in \delta^{+}\left(i\right)}\sum_{s\in \Omega_{ij}}X_{ijs}^{k}=1$, and according to Equation (15), there exists $\sum_{j\in \delta^{+}\left(i\right)}\sum_{s\in \Gamma_{ij}}X_{ijs}^{k}$ = 0); we are far more concerned about the unloading start time $l_{i}^{s}$ instead of $SA_{i}^{s}$, because the following operations cannot be conducted until $l_{i}^{s}$. Therefore, in such a case, $Y_{i}^{k}=l_{i}^{s}$, which is more effective than $Y_{i}^{k}=SA_{i}^{s}$.
(ii) If the following transportation is by rail service ($\sum_{j\in \delta^{+}\left(i\right)}\sum_{s\in \Gamma_{ij}}X_{ijs}^{k}=1$, and according to Equation (16), there exists $\sum_{j\in \delta^{+}\left(i\right)}\sum_{s\in \Omega_{ij}}X_{ijs}^{k}$=0), for similar reasons $Y_{i}^{k}=e_{i}^{s}$ instead of $SA_{i}^{s}$.

(25) $$Y_{d_{k}}^{k}\leq T_{k}\;\forall k\in K$$

Equation (25) is the due date constraint. It ensures that the arrival times of the flows of hazardous materials at their respective destinations should be later than the due dates claimed by customers.

(26) $$\left(\text{max}\left\{0,\;l_{i}^{s}-Y_{i}^{k}-\tau \right\}-Z_{ijs}^{k}\right)\cdot X_{ijs}^{k}=0\;\forall k\in K,\;\forall \left(i,j\right)\in A,\;\forall s\in \Gamma_{ij}$$

Equation (26) ensures the compatibility requirements among decision variables $X_{ijs}^{k}$, $Y_{i}^{k}$ and $Z_{ijs}^{k}$. It calculates the charged inventory times of the flow of each hazardous material at the nodes. Note that, when $X_{ijs}^{k}=0$, $\forall Z_{ijs}^{k}\geq 0$ all satisfy Equation (26), but the minimization of Component (12) in Objective 1 will finally restrict $Z_{ijs}^{k}=0$.

(27) $$W_{i}^{k}\in \left\{0,\;1\right\}\;\forall k\in K,\;\forall i\in N$$

(28) $$X_{ijs}^{k}\in \left\{0,\;1\right\}\;\forall k\in K,\;\forall \left(i,j\right)\in A,\;\forall s\in S_{ij}$$

(29) $$Y_{i}^{k}\geq 0\;\forall k\in K,\;\forall i\in N$$

(30) $$Z_{ijs}^{k}\geq 0\;\forall k\in K,\;\forall \left(i,j\right)\in A,\;\forall s\in \Gamma_{ij}$$

Equations (27)–(30) are the variable domain constraints.

## 5. Solution Strategy for the Bi-Objective Nonlinear Programming

### 5.1. Improved Linear Reformulation

The initial model built in Section 4.2 is easy to understand. However, due to the nonlinear Components (11) and (12) in Objective 1 and nonlinear Equations (21), (23), (24), and (26), which contain both multiplications of decision variables and the maximum function, this model is a typical nonlinear programming. These nonlinear functions make the model quite difficult to be solved by the exact solution algorithms. So, in this section, we design equivalent linear forms for the nonlinear components in order to reformulate the initial model. Then the model can be easily solved with the help of standard mathematical programming software, in which many exact solution algorithms can be effectively implemented. Although the equivalent linear forms are not very straightforward, the effectiveness of solving the reformulated mixed integer linear programming model is significantly improved. For detailed linear reformulation proofs, readers can refer to Appendix A: Model Linear Reformulation.

By replacing these nonlinear objective functions and constraints with the equivalent linear formulations given in Appendix A, the initial model is transformed to a mixed integer linear programming model shown as follows. We can then straightforwardly utilize the standard mathematical programming software (e.g., Lingo) to implement the exact solution algorithm (e.g., Branch-and-Bound Algorithm) to solve the model effectively.

- **Objective 1**
  $$\text{minimize }\underset{k\in K}{\sum }\underset{\left(i,j\right)\in A}{\sum }\underset{s\in S_{ij}}{\sum }c_{ijs}\cdot q_{k}\cdot X_{ijs}^{k}$$
  $$+\underset{k\in K}{\sum }\underset{i\in N}{\sum }\left(\underset{j\in \delta^{+}\left(i\right)}{\sum }\underset{s\in S_{ij}}{\sum }c_{s}\cdot q_{k}\cdot X_{ijs}^{k}+\underset{h\in \delta^{-}\left(i\right)}{\sum }\underset{r\in S_{hi}}{\sum }c_{r}\cdot q_{k}\cdot X_{hir}^{k}\right)-\underset{k\in K}{\sum }\underset{i\in N\backslash \left\{o_{k},\;d_{k}\;\right\}}{\sum }\left(2\cdot c_{\text{rail}}\cdot q_{k}\cdot U_{i}^{k}\right)$$
  $$+\underset{k\in K}{\sum }\underset{\left(i,j\right)\in A}{\sum }\underset{s\in \Gamma_{ij}}{\sum }c_{\text{store}}\cdot q_{k}\cdot V_{ijs}^{k}$$
- **Objective 2**
  $$\text{minimize }\underset{k\in K}{\sum }\underset{i\in N}{\sum }W_{i}^{k}\cdot POP_{i}\cdot q_{k}+\underset{k\in K}{\sum }\underset{\left(i,j\right)\in A}{\sum }\underset{s\in S_{ij}}{\sum }X_{ijs}^{k}\cdot POP_{ijs}\cdot q_{k}$$
- **Subject to**
  $$\underset{j\in \delta^{+}\left(i\right)}{\sum }\underset{s\in S_{ij}}{\sum }X_{ijs}^{k}-\underset{h\in \delta^{-}\left(i\right)}{\sum }\underset{r\in S_{hi}}{\sum }X_{hir}^{k}=\{\begin{array}{cc} 1, & i=o_{k}\\ 0, & \forall i\in N\backslash \left\{o_{k},\;d_{k}\;\right\}\\ -1, & i=d_{k} \end{array}\;\forall k\in K,\;\forall i\in N$$
  $$\underset{s\in S_{ij}}{\sum }X_{ijs}^{k}\leq 1\;\forall k\in K,\;\forall \left(i,\;j\right)\in A$$
  $$\underset{h\in \delta^{-}\left(i\right)}{\sum }\underset{r\in \Omega_{hi}}{\sum }X_{hir}^{k}+\underset{j\in \delta^{+}\left(i\right)}{\sum }\underset{s\in \Omega_{ij}}{\sum }X_{ijs}^{k}\leq 1\;\forall k\in K,\;\forall i\in N\backslash \left\{o_{k},\;d_{k}\;\right\}$$
  $$W_{i}^{k}=\{\begin{array}{c} \begin{array}{cc} \underset{j\in \delta^{+}\left(i\right)}{\sum }\underset{s\in S_{ij}}{\sum }X_{ijs}^{k}, & \forall i\in N\backslash \left\{o_{k},\;d_{k}\;\right\} \end{array}\\ \begin{array}{cc} 1, & i\in \left\{o_{k},\;d_{k}\;\right\} \end{array} \end{array}\;\forall k\in K,\;\forall i\in N$$
  $$U_{i}^{k}\leq \underset{h\in \delta^{-}\left(i\right)}{\sum }\underset{r\in \Gamma_{hi}}{\sum }X_{hir}^{k}+\underset{j\in \delta^{+}\left(i\right)}{\sum }\underset{s\in \Gamma_{ij}}{\sum }X_{ijs}^{k}-W_{i}^{k}\;\forall k\in K,\;\forall i\in N\backslash \left\{o_{k},\;d_{k}\;\right\}$$
  $$\underset{i\in N}{\sum }\underset{k\in K}{\sum }\frac{W_{i}^{k}\cdot q_{k}}{CAP_{i}}+\underset{k\in K}{\sum }\underset{\left(i,j\right)\in A}{\sum }\underset{s\in S_{ij}}{\sum }\frac{X_{ijs}^{k}\cdot q_{k}}{CAP_{ijs}}\leq ER_{\text{max}}$$
  $$\underset{k\in K}{\sum }q_{k}\cdot X_{ijs}^{k}\leq Q_{ijs}\;\forall \left(i,\;j\right)\in A,\;\forall s\in \Gamma_{ij}$$
  $$\begin{array}{c} Y_{i}^{k}\leq u_{i}^{s}\cdot \left(X_{ijs}^{k}+\underset{h\in \delta^{-}\left(i\right)}{\sum }\underset{s\in \Omega_{ij}}{\sum }X_{hir}^{k}-1\right)+\\ f_{i}^{s}\cdot \left(X_{ijs}^{k}+\underset{h\in \delta^{-}\left(i\right)}{\sum }\underset{s\in \Gamma_{ij}}{\sum }X_{hir}^{k}-1\right)+M\cdot \left(1-X_{ijs}^{k}\right) \end{array}\;\forall \left(i,\;j\right)\in A,\;\forall s\in \Gamma_{ij}$$
  $$Y_{o_{k}}^{k}=t_{\text{release}}^{k}\;\forall k\in K$$
  $$Y_{i}^{k}+t_{ijs}-Y_{j}^{k}\geq M\cdot \left(X_{ijs}^{k}-1\right)\;\forall k\in K,\;\forall \left(i,j\right)\in A,\;\forall s\in \Omega_{ij}$$
  $$Y_{i}^{k}+t_{ijs}-Y_{j}^{k}\leq M\cdot \left(1-X_{ijs}^{k}\right)\;\forall k\in K,\;\forall \left(i,j\right)\in A,\;\forall s\in \Omega_{ij}$$
  $$\begin{array}{c} l_{i}^{s}\cdot \underset{j\in \delta^{+}\left(i\right)}{\sum }\underset{s\in \Omega_{ij}}{\sum }X_{ijs}^{k}+e_{i}^{s}\cdot \underset{j\in \delta^{+}\left(i\right)}{\sum }\underset{s\in \Gamma_{ij}}{\sum }X_{ijs}^{k}-Y_{i}^{k}\geq \\ M\cdot \left(X_{hir}^{k}-1\right) \end{array}\;\forall k\in K,\;\forall \left(h,i\right)\in A,\;\forall r\in \Gamma_{hi}$$
  $$\begin{array}{c} l_{i}^{s}\cdot \underset{j\in \delta^{+}\left(i\right)}{\sum }\underset{s\in \Omega_{ij}}{\sum }X_{ijs}^{k}+e_{i}^{s}\cdot \underset{j\in \delta^{+}\left(i\right)}{\sum }\underset{s\in \Gamma_{ij}}{\sum }X_{ijs}^{k}-Y_{i}^{k}\leq \\ M\cdot \left(1-X_{hir}^{k}\right) \end{array}\;\forall k\in K,\;\forall \left(h,i\right)\in A,\;\forall r\in \Gamma_{hi}$$
  $$Z_{ijs}^{k}\geq M\cdot \left(X_{ijs}^{k}-1\right)+\left(l_{i}^{s}-Y_{i}^{k}-\tau \right)\;\forall k\in K,\;\forall \left(i,j\right)\in A,\;\forall s\in \Gamma_{ij}$$
  $$Z_{ijs}^{k}\leq M\cdot X_{ijs}^{k}\;\forall k\in K,\;\forall \left(i,j\right)\in A,\;\forall s\in \Gamma_{ij}$$
  $$V_{ijs}^{k}\geq Z_{ijs}^{k}+M\cdot \left(\underset{h\in \delta^{-}\left(i\right)}{\sum }\underset{r\in \Omega_{ij}}{\sum }X_{hir}^{k}-1\right)\;\forall k\in K,\;\forall \left(i,j\right)\in A,\;\forall s\in \Gamma_{ij}$$
  $$U_{i}^{k}\geq 0\;\forall k\in K,\;\forall i\in N\backslash \left\{o_{k},\;d_{k}\;\right\}$$
  $$V_{ijs}^{k}\geq 0\;\forall k\in K,\;\forall \left(i,j\right)\in A,\;\forall s\in \Gamma_{ij}$$
  $$W_{i}^{k}\in \left\{0,\;1\right\}\;\forall k\in K,\;\forall i\in N$$
  $$X_{ijs}^{k}\in \left\{0,\;1\right\}\;\forall k\in K,\;\forall \left(i,j\right)\in A,\;\forall s\in S_{ij}$$
  $$Y_{i}^{k}\geq 0\;\forall k\in K,\;\forall i\in N$$
  $$Z_{ijs}^{k}\geq 0\;\forall k\in K,\;\forall \left(i,j\right)\in A,\;\forall s\in \Gamma_{ij}$$

### 5.2. Normalized Weighted Sum Method for the Bi-Objective Optimization

**Figure 1:**
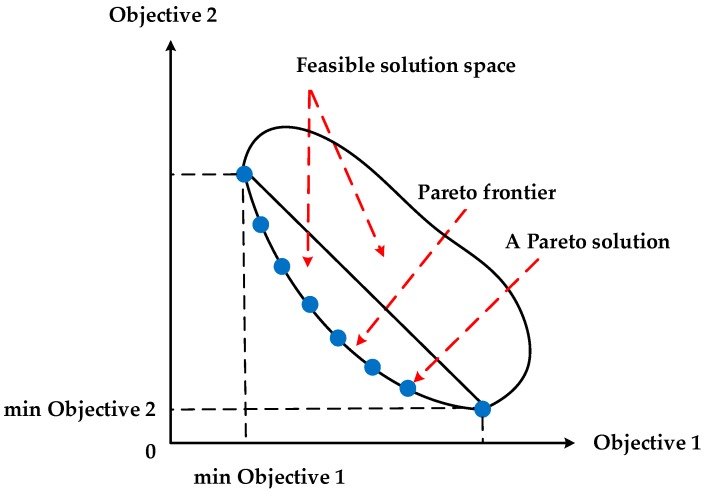
An example of the Pareto solutions to a bi-objective optimization.

Multi-objective optimization problems have two kinds of solutions, dominated solutions and Pareto solutions (non-dominated). For the multi-objective optimization with dominated solutions, its multiple objectives will reach their respective optimum simultaneously, i.e., the optimal solution for a certain objective is also the optimal one for the remaining objectives. Consequently, there only exists one optimal solution for such a multi-objective optimization. However, in most cases, the objectives in the multi-objective optimization are in mutual conflict, and the objectives cannot reach their optimum simultaneously, i.e., the respective optimal solutions for the objectives are different. There usually exists a group of solutions to such multi-objective optimization, namely Pareto solutions or non-dominated solutions. A group of Pareto solutions forms the Pareto frontier of the multi-objective optimization problem. An example of the Pareto solutions to the multi-objective optimization can be seen in Figure 1 [32]. In multi-objective decision making, with the help of the Pareto frontier of the problem, decision-makers can make a trade-off between different objectives.

There are many methods that can be utilized to generate Pareto solutions to a multi-objective optimization problem. One of the most classical and extensively used methods is the weighted sum method. In this method, different weights are distributed to the objectives, and then the weighted objectives are combined linearly to transform the multi-objective optimization problem into a single-objective one. It is a simple method that is easy to understand and utilize. Hence it has already received wide application, e.g., Xie et al. [22], Sheu [33], Samanlioglu [34], and Rakas et al. [35]. Specifically for the bi-objective optimization model constructed in this study, after weighted summation, there are:

(31) $$\text{minimize }\lambda_{1}\cdot f_{1}+\lambda_{2}\cdot f_{2}$$

(32) $$\lambda_{1}+\lambda_{2}=1$$

(33) $$0\leq \lambda_{1}\leq 1$$

(34) $$0\leq \lambda_{2}\leq 1$$

where $f_{1}$ and $f_{2}$ represent the objective functions of minimizing the generalized costs and of minimizing the social risk along the planned routes, $\lambda_{1}$ and $\lambda_{2}$ are the distributed weights to the two objectives, and their values are determined by decision makers before model simulation. We can gain different solutions when different values are assigned to $\lambda_{1}$ and $\lambda_{2}$, and finally generate the Pareto frontier for the problem.

In the classical weighted sum method, the objectives usually have different units and magnitudes, which will affect the optimization result when directly utilizing this method to address the multi-objective optimization problem. Consequently, in this study, we adopt an improved weighted sum method, namely the normalized weighted sum method, to solve our bi-objective optimization problem.

The normalized weighted sum method first solves the multi-objective optimization problem as a series of single-objective ones, and obtains their respective optimal solution. For the specific model in this study, let $f_{1}^{*}$ and $f_{2}^{*}$ separately denote the optimal values of Objective 1 and of Objective 2. After dividing by their respective optimal values, the objectives become normalized and there is no difference regarding the unit and magnitude among the different objectives. Therefore, in the normalized weighted sum method, the objective function is as below instead of as in Equation (31):

(35) $$\text{minimize }\lambda_{1}\cdot \frac{f_{1}}{f_{1}^{*}}+\lambda_{2}\cdot \frac{f_{2}}{f_{2}^{*}}$$

By using the normalized weighted sum method introduced in this section, we can generate the Pareto frontier for the case that will be discussed in the next section.

## 6. Empirical Case Study from the Beijing–Tianjin–Hebei Region in China

### 6.1. Case Description and Parameter Setting

**Figure 2:**
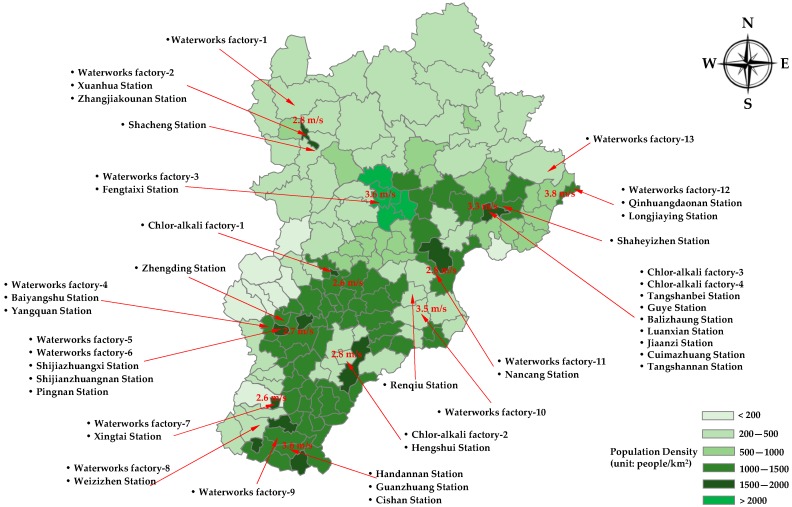
Distribution of the nodes in the empirical case.

In this section, we design an empirical case regarding liquefied chlorine transportation to demonstrate the feasibility of the proposed methods in dealing with a practical problem. The empirical case is based on a Beijing–Tianjin–Hebei Region scenario in China, in which four chlorine–alkali chemical factories in Hebei Province supply liquefied chlorine to several waterworks factories in the Beijing–Tianjin–Hebei Region. The locations of the nodes in the region are shown in Figure 2. The population density distribution and average wind speed distribution of the region are also illustrated in Figure 2. They can be used to estimate the population exposure in the empirical case.

In the large-scale empirical case, we assume that the liquefied chlorine is carried in 40-ft tank containers (in Equation (2), VOL = 30,480 kg). The most common consequence in the liquefied chlorine transportation accidents is that the liquefied chlorine constantly releases from the damaged tank container and disperses into the surroundings as gas. This kind of accident accounts for 95% of total liquefied chlorine transportation accidents [36]. In such a transportation accident, the release of chlorine usually lasts about one hour (in Equation (2), T = 3600 s) [37]. Therefore, according to Equation (2), when evaluating the population exposure, the release rate Q = 8.5 kg/s (8.5 × 10^6^ mg/s). The IDLH concentration threshold $\bar{C}$ in Equations (4) and (5) is 2500 mg/m^3^ [37]. Considering the conservative evaluation of the social risk, the atmospheric stability category is set to Level D. Consequently, in Equations (4) and (5), a = 0.15, b = 0.89, c = 0.40, and d = 0.63. Based on the information provided above, we can obtain the population exposure and environmental capacity of each node shown in Appendix B, Table B1. When evaluating the environmental capacity, in Equations (6) and (7), the allowed emissions concentration threshold $C^{*}$ is 80 mg/m^3^ according to GB 16297-1996, and the potential impact radius $R^{*}\;$= 8 km [38]. Therefore, the environmental capacity of each node is 85,743 tons.

**Figure 3:**
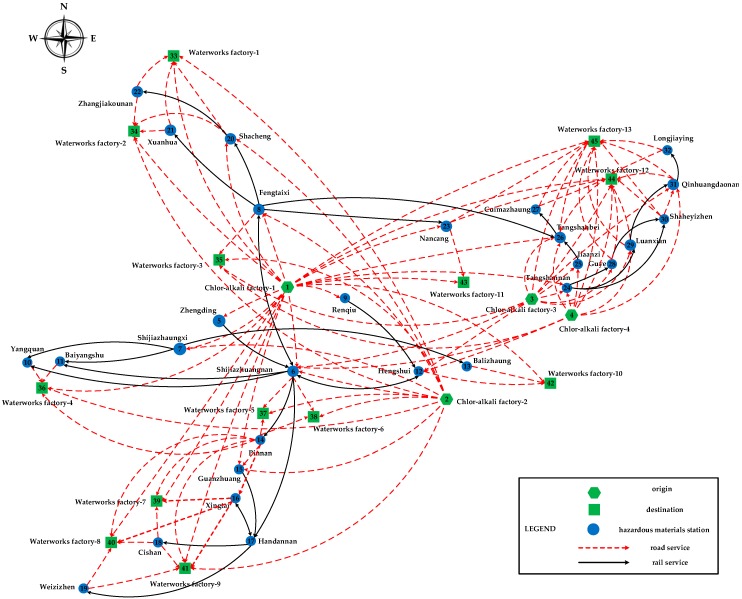
Topological structure of the multimodal service network for the empirical case.

The detailed topological structure of the multimodal service network for this empirical case is shown as Figure 3. It is composed of 45 nodes (including four origin nodes, 13 destination nodes, and 28 hazardous materials station nodes) and 132 directed arcs (including 31 rail service arcs and 101 road service arcs). The corresponding information on the road services in the multimodal service network is presented in Table B2. The schedules of the rail services in the multimodal service network as well as their risk parameters are given in Table B3. In Table B3, all the time data are discretized into real numbers (e.g., 10:30 on the first day is transformed into 10.5; on the second day, it is transformed into 34.5), and the same rail services operated in different periods are treated as different ones. In addition, the operation periods of all the rail services are one day/train.

The cost parameters in the road–rail multimodal routing problem of hazardous materials are stated as follows. For the rail service, its transportation unit costs can be determined by $c_{ijs}=c_{\text{rail}}^{1}+c_{\text{rail}}^{2}\cdot d_{ijs}$, where $c_{\text{rail }}^{1}$= 27.9 ¥/ton and $c_{\text{rail }}^{2}$= 0.206 ¥/ton-km. Its unit loading/unloading costs $c_{\text{rail}}\;$= 5.8 ¥/ton. The unit inventory costs $c_{\text{store}}\;$= 0.1 ¥/ton-h. The free-of-charge inventory period is 48 h. For the road service, its transportation unit costs can be determined by $c_{ijs}=c_{\text{road}}^{2}\cdot d_{ijs}$, where $c_{\text{road}}^{2}\;$= 0.76 ¥/ton-km. Its unit loading/unloading costs $c_{\text{road}}$ = 5.5 ¥/ton. In this empirical case, there are 25 hazardous material flows that need to be routed. Their origins, destinations, volumes, release times, and due dates are all listed in Table 1. In Table 1, the release times and due dates are all real numbers.

### 6.2. Optimization Results and Discussions

#### 6.2.1. Simulation Environment

We set the environmental risk threshold $ER_{\text{max}}\;$= 0.6. Then this empirical case, which is formulated as a linear programming problem, can be solved by the exact solution algorithms. In this study, we utilize the Branch-and-Bound Algorithm to solve the specific routing problem. This algorithm is implemented by the mathematical programming software Lingo 12 (LINDO Systems Inc., Chicago, IL, USA) on a ThinkPad Laptop with Intel Core i5-5200U 2.20 GHz CPU 8 GB RAM. The scale of the empirical case problem is indicated in Table 2.

#### 6.2.2. Multimodal Routes Illustration

First of all, we use the optimal routes with minimal generalized costs as examples to indicate how to transport hazardous materials in the road–rail multimodal service network. In this empirical case, the least-costs road–rail multimodal routes are presented in Table 3, where items such as #40103 and #32123 represent the train numbers of the rail services. As we can see from Table 3, there are two kinds of routes for the transportation of hazardous materials in the multimodal service network, including single road service routes and road–rail multimodal routes. In the latter, rail services are dominant, and road services are helpful supplements to realize the through transportation from shippers to receivers. Such routes are quite suitable for transporting hazardous materials with longer lead times.

**Figure 4:**
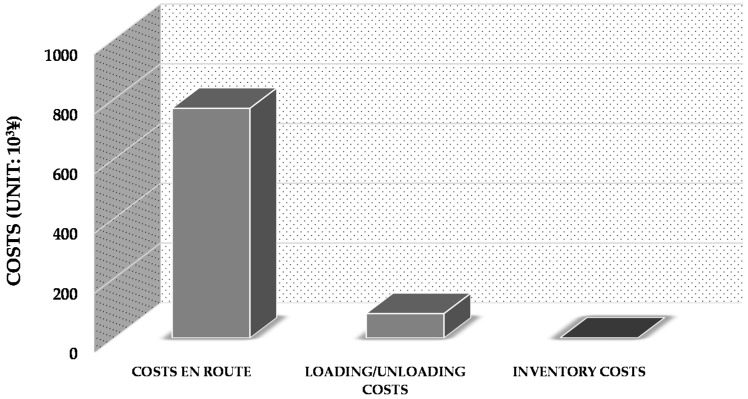
Structure of the minimal generalized costs.

The minimal generalized costs are 850,192 ¥ in this empirical case. The structure of these costs is shown in Figure 4. As we can see from Figure 4, costs en route account for the majority of the total generalized costs (in this case, approximately 90%). The planning horizon of the empirical case is limited; the inventory times of the hazardous materials are hence shorter than the free-of-charge inventory period. Consequently, as for this empirical case, there are no inventory costs in the minimal generalized costs.

#### 6.2.3. Single-Objective Scenarios

First we address the two single-objective optimization problems that separately aim at minimizing the total generalized costs (unit: ¥) and the social risk (unit: 10^4^ people-ton). The respective optimal solutions to the three objectives are given in Table 4. Table 4 clearly indicates that the two objectives cannot reach their respective optimal values simultaneously, i.e., the optimal solution to a certain objective is not the optimal one for the remaining objective. Therefore, there exist Pareto solutions for the bi-objective optimization problem.

#### 6.2.4. Bi-Objective Scenario

**Figure 5:**
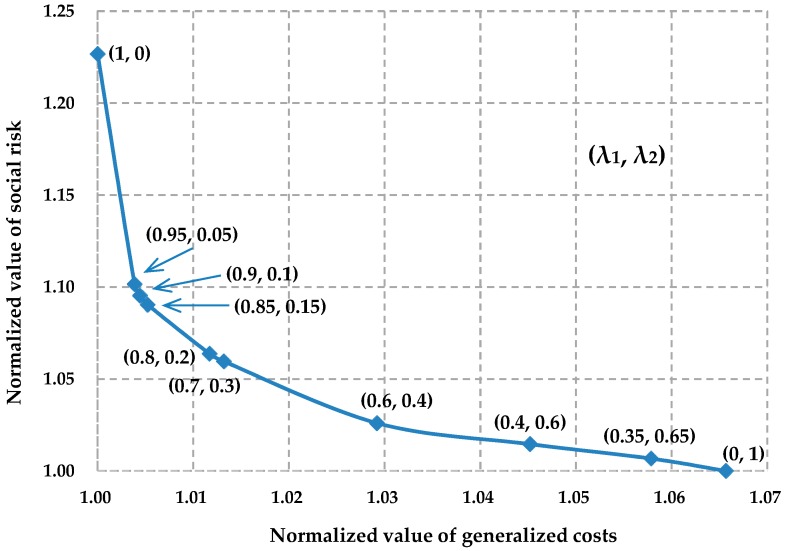
Pareto solutions to the bi-objective optimization problem of the empirical case.

In bi-objective decision making, because the two objectives are conflicting, decision-makers should make a trade-off between lowering the generalized costs and reducing the social risk. By using the normalized weighted sum method introduced in Section 5, we can generate Pareto solutions (Pareto frontier) to the bi-objective optimization problem as in Figure 5. Figure 5 shows that improving the economy of the multimodal routing will lead to an increase in the social risk along the planned routes, and reducing the social risk along the planned routes will lower the economy of the multimodal routing.

Figure 5 provides various candidate solutions to the bi-objective optimization problem that will be helpful for decision-makers trying to balance the conflict between cost objectives and risk objectives. For example, in the trade-off between generalized costs and social risk, if decision-makers consider that it is acceptable as long as the normalized social risk is lower than 1.05, then the last four Pareto solutions in Figure 5 can be their candidate multimodal route schemes. Considering minimization of the generalized costs, decision-makers will finally select the multimodal route corresponding to the 7th Pareto solution to transport the hazardous materials.

#### 6.2.5. Sensitivity of the Pareto Solutions with Respect to the Environmental Risk Threshold

**Figure 6:**
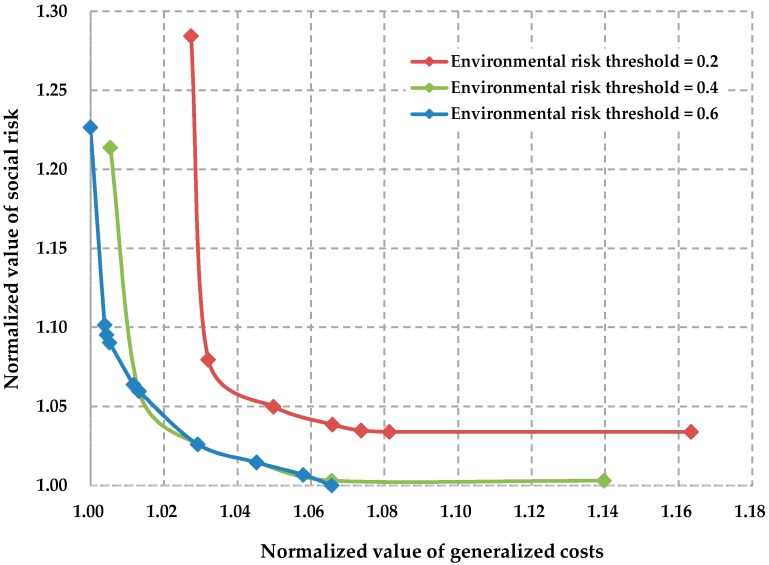
Sensitivity of the Pareto frontier with respect to the environmental risk threshold.

In this section, we conduct sensitivity analysis of the Pareto solutions with respect to the environmental risk threshold. Because the minimal environmental risk in the multimodal service network is 0.135, we generate three Pareto frontiers corresponding to the thresholds 0.2, 0.4, and 0.6. Figure 6 shows the variation of the Pareto frontiers when the environmental risk threshold increases. In Figure 6, the generalized costs and social risk are separately normalized by 850,192 and 506,362, which are the optimal values of generalized costs and social risk, respectively, when the environmental risk threshold is 0.6.

As we can see from Figure 6, the Pareto frontier of the bi-objective optimization problem will move to the left with the increase of the environmental risk threshold. That is to say, we can lower the generalized costs and the social risk simultaneously if we relax the environmental risk constraint by enlarging the environmental risk threshold. The extrapolated Figure 6 can also provide a reference for decision-makers when they are not sure of the setting of the environmental risk threshold for the multimodal service network.

## 7. Conclusions

In this study, we explore the road–rail multimodal routing problem of hazardous materials from the operational level of network planning. The main contribution made in this study is its comprehensive formulation of the following characteristics, so that the specific routing problem can be addressed from an improved practical viewpoint: (1) specific customer demands from transportation economy and efficiency; (2) multiple hazardous materials flows; (3) railway schedule-based space–time constraints; (4) environmental risk constraint; and (5) bi-objective optimization from the viewpoint of generalized costs vs. social risk management. Consideration of all these formulation characteristics significantly enhances the feasibility of the proposed model in dealing with practical problems, which is also a good development of current studies on the routing problem of hazardous materials.

Besides the improvement in model formulation, this study also develops concise linear reformulations that transform the initial nonlinear model into its equivalent linear programming. On the one hand, the linear reformulations enable the problem to be solved effectively by an exact solution algorithm that can be easily implemented by standard mathematical programming software. On the other hand, the linearization method can also provide an exact benchmark in order to systematically test the performance of various heuristic algorithms in dealing with the specific routing problem. In addition, the exact solution algorithm, e.g., the Branch-and-Bound Algorithm, can also test whether the model itself is mathematically logical or not.

In this large-scale empirical case study, by using the Branch-and-Bound Algorithm in Lingo 12, the optimal solution for a certain scenario can be generated within 3 min. Although the computational time for a single solution is acceptable, a large amount of computational time will be consumed when several Pareto solutions are required and a sensitivity analysis needs to be conducted. Therefore, it is still necessary to design a heuristic algorithm to address the problem more efficiently. Consequently, our further study will focus on the design of the heuristic algorithm. As claimed above, this study has provided an exact benchmark for testing the heuristic algorithm, which has provided a solid foundation for us to carry on such a study.

## Figures and Tables

**Table 1:** Information on the hazardous materials flows in the large-scale empirical example.

No.	Origin	Destination	Volume (Unit: Ton)	Release Time	Due Date
1	1	33	270	0	9.5
2	1	34	120	8	14
3	1	36	60	5	26
4	1	39	180	14	40
5	1	40	120	6	25
6	1	41	90	14	39
7	1	42	120	28	47
8	1	43	210	32.5	40
9	1	44	270	24.5	48
10	1	45	90	1	25
11	2	33	60	0	58
12	2	34	180	25	60
13	2	35	270	8	19
14	2	36	250	13.9	39
15	2	37	90	21.5	45.5
16	2	38	180	27	41
17	2	41	210	30	42
18	3	35	150	3.2	19
19	3	44	120	23	41
20	3	44	210	28	42
21	3	45	180	8.5	23
22	4	35	120	18	30.5
23	4	44	210	12.5	31
24	4	45	240	0	9
25	4	45	270	5	15

**Table 2:** Scale of the empirical case problem.

Total Variables	Integer Variables	Constraints
22,403	8925	60,006

**Table 3:** Multimodal routes with minimal generalized costs for the large-scale empirical case.

No.	Multimodal Routes	Arrival Time at Destination
1	**1** $\vec{\text{road service}}$ **33**	5
2	**1** $\vec{\text{road service}}$ **34**	11.3
3	**1** $\vec{\text{road service}}$ **36**	8.2
4	**1** $\vec{\text{road service}}$ **9** $\vec{\#40103}$ **12** $\vec{\#\;30003}$ **16** $\vec{\text{road service}}$ **39**	36
5	**1** $\vec{\text{road service}}$ **6** $\vec{\#\;32123}$ **17** $\vec{\#\;39101}$ **18** $\vec{\text{road service}}$ **40**	23
6	**1** $\vec{\text{road service}}$ **9** $\vec{\#\;40103}$ **12** $\vec{\#\;30003}$ **16** $\vec{\text{road service}}$ **41**	37.3
7	**1** $\vec{\text{road service}}$ **42**	30.3
8	**1** $\vec{\text{road service}}$ **43**	34.8
9	**1** $\vec{\text{road service}}$ **8** $\vec{\#\;38027}$ **26** $\vec{\text{road service}}$ **44**	47.6
10	**1** $\vec{\text{road service}}$ **8** $\vec{\#\;38001}$ **26** $\vec{\text{road service}}$ **45**	24.5
11	2 $\vec{\text{road service}}$ **6** $\vec{\#\;21018}$ **8** $\vec{\#\;33057}$ **20** $\vec{\#\;33203}$ **22** $\vec{\text{road service}}$ **33**	8.7
12	**2** $\vec{\text{road service}}$ **8** $\vec{\#\;33067}$ **21** $\vec{\text{road service}}$ **34**	45.1
13	**2** $\vec{\text{road service}}$ **35**	11
14	**2** $\vec{\text{road service}}$ **36**	16.9
15	**2** $\vec{\text{road service}}$ **37**	23.4
16	**2** $\vec{\text{road service}}$ **38**	28.8
17	**2** $\vec{\text{road service}}$ **12** $\vec{\#\;30003}$ **16** $\vec{\text{road service}}$ **41**	37.3
18	**3** $\vec{\text{road service}}$ **35**	5.9
19	**3** $\vec{\text{road service}}$ **29** $\vec{\#\;43093}$ **31** $\vec{\text{road service}}$ **44**	39.1
20	**3** $\vec{\text{road service}}$ **29** $\vec{\#\;43093}$ **31** $\vec{\text{road service}}$ **44**	39.1
21	**3** $\vec{\text{road service}}$ **45**	10.8
22	**4** $\vec{\text{road service}}$ **35**	20.8
23	**4** $\vec{\text{road service}}$ **44**	14.4
24	**4** $\vec{\text{road service}}$ **45**	2.8
25	**4** $\vec{\text{road service}}$ **45**	7.8

**Table 4:** Optimization results of the three single-objective scenarios.

Performance	Scenario 1: Min Objective 1	Scenario 2: Min Objective 2
Objective 1	850,192 *	906,037
Objective 2	621,099	506,362 *
Environmental risk	0.553	0.459
Solver state	Global opt	Global opt
Computational time	2 min 51 s **	2 min 38 s **

* optimal values of the objectives; ** average value of 10 simulations.

## Appendix A. Model Linear Reformulation

- **Reformulation 1.** By using a non-negative variable $U_{i}^{k}$ to replace the nonlinear function $\text{max}\left\{\sum_{h\in \delta^{-}\left(i\right)}\sum_{r\in \Gamma_{hi}}X_{hir}^{k}+\sum_{j\in \delta^{+}\left(i\right)}\sum_{s\in \Gamma_{ij}}X_{ijs}^{k}-1,\;0\right\}$ and adding two linear Equations (A2) and (A3), nonlinear Component (11) in Objective 1 can be reformulated as a linear function (A1).
  (A1) $$\underset{k\in K}{\sum }\underset{i\in N}{\sum }\left(\underset{j\in \delta^{+}\left(i\right)}{\sum }\underset{s\in S_{ij}}{\sum }c_{s}\cdot q_{k}\cdot X_{ijs}^{k}+\underset{h\in \delta^{-}\left(i\right)}{\sum }\underset{r\in S_{hi}}{\sum }c_{r}\cdot q_{k}\cdot X_{hir}^{k}\right)-\underset{k\in K}{\sum }\underset{i\in N\backslash \left\{o_{k},\;d_{k}\;\right\}}{\sum }\left(2\cdot c_{\text{rail}}\cdot q_{k}\cdot U_{i}^{k}\right)$$
  (A2) $$U_{i}^{k}\leq \underset{h\in \delta^{-}\left(i\right)}{\sum }\underset{r\in \Gamma_{hi}}{\sum }X_{hir}^{k}+\underset{j\in \delta^{+}\left(i\right)}{\sum }\underset{s\in \Gamma_{ij}}{\sum }X_{ijs}^{k}-W_{i}^{k}\;\forall k\in K,\;\forall i\in N\backslash \left\{o_{k},\;d_{k}\;\right\}$$
  (A3) $$U_{i}^{k}\geq 0\;\forall k\in K,\;\forall i\in N\backslash \left\{o_{k},\;d_{k}\;\right\}$$

$U_{i}^{k}$ has the same function of $\text{max}\left\{\sum_{h\in \delta^{-}\left(i\right)}\sum_{r\in \Gamma_{hi}}X_{hir}^{k}+\sum_{j\in \delta^{+}\left(i\right)}\sum_{s\in \Gamma_{ij}}X_{ijs}^{k}-1,\;0\right\}$. If and only if $U_{i}^{k}\;$= 1, there ought to be no loading and unloading costs for hazardous materials k at node i, and the extra loading and unloading costs contained in $\sum_{k\in K}\sum_{i\in N}\left(\sum_{j\in \delta^{+}\left(i\right)}\sum_{s\in S_{ij}}c_{s}\cdot q_{k}\cdot X_{ijs}^{k}+\sum_{h\in \delta^{-}\left(i\right)}\sum_{r\in S_{hi}}c_{r}\cdot q_{k}\cdot X_{hir}^{k}\right)$ should be deducted. The following explanation proves that $U_{i}^{k}$ is equivalent to $\text{max}\left\{\sum_{h\in \delta^{-}\left(i\right)}\sum_{r\in \Gamma_{hi}}X_{hir}^{k}+\sum_{j\in \delta^{+}\left(i\right)}\sum_{s\in \Gamma_{ij}}X_{ijs}^{k}-1,\;0\right\}$.

There are three independent cases in Component (11):

(i) Hazardous materials k get transshipped between rail services at node i, i.e., $\sum_{h\in \delta^{-}\left(i\right)}\sum_{r\in \Gamma_{hi}}X_{hir}^{k}$ = 1 and $\sum_{j\in \delta^{+}\left(i\right)}\sum_{s\in \Gamma_{ij}}X_{ijs}^{k}$ = 1, in this case, $\text{max}\left\{\sum_{h\in \delta^{-}\left(i\right)}\sum_{r\in \Gamma_{hi}}X_{hir}^{k}+\sum_{j\in \delta^{+}\left(i\right)}\sum_{s\in \Gamma_{ij}}X_{ijs}^{k}-1,\;0\right\}\;$= 1.
(ii) Hazardous materials k get transshipped between road service and rail service at node i, i.e., $\sum_{h\in \delta^{-}\left(i\right)}\sum_{r\in \Gamma_{hi}}X_{hir}^{k}\;$=1 and $\sum_{j\in \delta^{+}\left(i\right)}\sum_{s\in \Gamma_{ij}}X_{ijs}^{k}$ = 0 or $\sum_{h\in \delta^{-}\left(i\right)}\sum_{r\in \Gamma_{hi}}X_{hir}^{k}$ = 0 and $\sum_{j\in \delta^{+}\left(i\right)}\sum_{s\in \Gamma_{ij}}X_{ijs}^{k}\;$= 1, in this case, $\text{max}\left\{\sum_{h\in \delta^{-}\left(i\right)}\sum_{r\in \Gamma_{hi}}X_{hir}^{k}+\sum_{j\in \delta^{+}\left(i\right)}\sum_{s\in \Gamma_{ij}}X_{ijs}^{k}-1,\;0\right\}\;$= 0.
(iii) Node i is not covered in the route of hazardous materials k, i.e., $\sum_{h\in \delta^{-}\left(i\right)}\sum_{r\in \Gamma_{hi}}X_{hir\;}^{k}$= 0 and $\sum_{j\in \delta^{+}\left(i\right)}\sum_{s\in \Gamma_{ij}}X_{ijs}^{k}\;$= 0, in this case, $\text{max}\left\{\sum_{h\in \delta^{-}\left(i\right)}\sum_{r\in \Gamma_{hi}}X_{hir}^{k}+\sum_{j\in \delta^{+}\left(i\right)}\sum_{s\in \Gamma_{ij}}X_{ijs}^{k}-1,\;0\right\}\;$= 0.

For Case (i), obviously, $W_{i}^{k}\;$= 1, and $U_{i}^{k}\leq 1$ according to Equation (A2). Then Equation (A3) and the minimization of Component (A1) will restrict $U_{i}^{k}\;$= 1. For Case (ii), $W_{i}^{k}\;$= 1 also exists, and $U_{i}^{k}\leq 0$ according to Equation (A2). Due to the restriction of Equation (A3), $U_{i}^{k}\;$= 0. For Case (iii), $W_{i}^{k}\;$= 0, similarly to Case ii, $U_{i}^{k}$ will finally equal to 0. Above all, when inputting the same cases into Equations (32)~(34), $U_{i}^{k}$ will contribute the same results to $\text{max }\left\{\sum_{h\in \delta^{-}\left(i\right)}\sum_{r\in \Gamma_{hi}}X_{hir}^{k}+\sum_{j\in \delta^{+}\left(i\right)}\sum_{s\in \Gamma_{ij}}X_{ijs}^{k}-1,\;0\right\}$. Consequently, the equivalency above is verified.

- **Reformulation 2.** By using a non-negative decision variable $V_{ijs}^{k}$ to replace the nonlinear function $Z_{ijs}^{k}\cdot \sum_{h\in \delta^{-}\left(i\right)}\sum_{r\in \Omega_{ij}}X_{hir}^{k}$ and adding two linear Equations (A5) and (A6), the nonlinear Component (12) in Objective 1 can be reformulated as a linear function (A4).
  (A4) $$\underset{k\in K}{\sum }\underset{\left(i,j\right)\in A}{\sum }\underset{s\in \Gamma_{ij}}{\sum }c_{\text{store}}\cdot q_{k}\cdot V_{ijs}^{k}$$
  (A5) $$V_{ijs}^{k}\geq Z_{ijs}^{k}+M\cdot \left(\underset{h\in \delta^{-}\left(i\right)}{\sum }\underset{r\in \Omega_{ij}}{\sum }X_{hir}^{k}-1\right)\;\forall k\in K,\;\forall \left(i,j\right)\in A,\;\forall s\in \Gamma_{ij}$$
  (A6) $$V_{ijs}^{k}\geq 0\;\forall k\in K,\;\forall \left(i,j\right)\in A,\;\forall s\in \Gamma_{ij}$$

In this reformulation, the following two cases obviously exist, which proves the equivalency above.

(i) If $\sum_{h\in \delta^{-}\left(i\right)}\sum_{r\in \Omega_{ij}}X_{hir}^{k}$ = 0, i.e., hazardous materials k arrive at node i by the rail service, there are $V_{ijs}^{k}\geq -M$ and $V_{ijs}^{k}\geq 0$ according to Equations (A5) and (A6), and the minimization of Component (A4) will restrict $V_{ijs}^{k}\;$= 0, which matches the setting that there are no inventory costs at the node if the hazardous materials are transshipped between different rail services.
(ii) If $\sum_{h\in \delta^{-}\left(i\right)}\sum_{r\in \Omega_{ij}}X_{hir}^{k}$ = 1, i.e., hazardous materials k arrive at node i by road service, there are $V_{ijs}^{k}\geq Z_{ijs}^{k}$ and $V_{ijs}^{k}\geq 0$, and minimization of Component (A4) will restrict $V_{ijs}^{k}=Z_{ijs}^{k}$, which matches the setting that inventory costs will be charged at the node according to the charged inventory time if the hazardous materials are transshipped from road service to rail service.

- **Reformulation 3.** Nonlinear Equation (21) is equivalent to linear Equation (A7) as follows.
  (A7) $$\begin{array}{c} Y_{i}^{k}\leq u_{i}^{s}\cdot \left(X_{ijs}^{k}+\underset{h\in \delta^{-}\left(i\right)}{\sum }\underset{s\in \Omega_{ij}}{\sum }X_{hir}^{k}-1\right)+\\ f_{i}^{s}\cdot \left(X_{ijs}^{k}+\underset{h\in \delta^{-}\left(i\right)}{\sum }\underset{s\in \Gamma_{ij}}{\sum }X_{hir}^{k}-1\right)+M\cdot \left(1-X_{ijs}^{k}\right) \end{array}\;\forall \left(i,\;j\right)\in A,\;\forall s\in \Gamma_{ij}$$

It is obvious that we can gain the same results as Equation (21) after we separately input the three given cases, Case (i)–Case (iii), from Equation (21) into Equation (A7). Therefore, the two constraints are equivalent to each other.

- **Reformulation 4.** Nonlinear Equations (23), (24) and (26) are separately equivalent to linear Equations (A8)–(A13). For detailed proofs of the above three equivalencies, readers can refer to our previous study [26].
  (A8) $$Y_{i}^{k}+t_{ijs}-Y_{j}^{k}\geq M\cdot \left(X_{ijs}^{k}-1\right)\;\forall k\in K,\;\forall \left(i,j\right)\in A,\;\forall s\in \Omega_{ij}$$
  (A9) $$Y_{i}^{k}+t_{ijs}-Y_{j}^{k}\leq M\cdot \left(1-X_{ijs}^{k}\right)\;\forall k\in K,\;\forall \left(i,j\right)\in A,\;\forall s\in \Omega_{ij}$$
  (A10) $$\begin{array}{c} l_{i}^{s}\cdot \underset{j\in \delta^{+}\left(i\right)}{\sum }\underset{s\in \Omega_{ij}}{\sum }X_{ijs}^{k}+e_{i}^{s}\cdot \underset{j\in \delta^{+}\left(i\right)}{\sum }\underset{s\in \Gamma_{ij}}{\sum }X_{ijs}^{k}-Y_{i}^{k}\geq \\ M\cdot \left(X_{hir}^{k}-1\right) \end{array}\;\forall k\in K,\;\forall \left(h,i\right)\in A,\;\forall r\in \Gamma_{hi}$$
  (A11) $$\begin{array}{c} l_{i}^{s}\cdot \underset{j\in \delta^{+}\left(i\right)}{\sum }\underset{s\in \Omega_{ij}}{\sum }X_{ijs}^{k}+e_{i}^{s}\cdot \underset{j\in \delta^{+}\left(i\right)}{\sum }\underset{s\in \Gamma_{ij}}{\sum }X_{ijs}^{k}-Y_{i}^{k}\leq \\ M\cdot \left(1-X_{hir}^{k}\right) \end{array}\;\forall k\in K,\;\forall \left(h,i\right)\in A,\;\forall r\in \Gamma_{hi}$$
  (A12) $$Z_{ijs}^{k}\geq M\cdot \left(X_{ijs}^{k}-1\right)+\left(l_{i}^{s}-Y_{i}^{k}-\tau \right)\;\forall k\in K,\;\forall \left(i,j\right)\in A,\;\forall s\in \Gamma_{ij}$$
  (A13) $$Z_{ijs}^{k}\leq M\cdot X_{ijs}^{k}\;\forall k\in K,\;\forall \left(i,j\right)\in A,\;\forall s\in \Gamma_{ij}$$

## Appendix B

**Table B1:** Population exposures around the nodes in the empirical case.

Node	Population Exposure (Unit: 10^4^ People)	Node	Population Exposure (Unit: 10^4^ People)	Node	Population Exposure (Unit: 10^4^ People)
1	3.44	16	2.87	31	1.74
2	0.66	17	1.32	32	1.74
3	2.29	18	0.58	33	0.50
4	2.29	19	0.58	34	2.48
5	2.31	20	0.66	35	3.56
6	2.13	21	2.48	36	2.13
7	2.84	22	2.48	37	2.84
8	3.56	23	2.32	38	2.84
9	0.32	24	2.29	39	2.87
10	2.13	25	2.29	40	0.58
11	2.13	26	2.29	41	1.12
12	0.66	27	2.29	42	0.43
13	2.29	28	2.29	43	2.32
14	2.13	29	2.29	44	1.74
15	1.32	30	1.69	45	0.82

**Table B2:** Transportation distances, times, and risk parameters for road services in the large-scale empirical example.

Arc	Distance (Unit: km)	Time (Unit: h)	Population Exposure (Unit: 10^4^ People)	Environmental Capacity (Unit: 10^4^ Ton)	Arc	Distance (Unit: km)	Time (Unit: h)	Population Exposure (Unit: 10^4^ People)	Environmental Capacity (Unit: 10^4^ Ton)
(1, 5)	129	1.8	57.17	7.47	(4, 26)	80	1	56.72	4.64
(1, 6)	153	2.3	81.36	8.86	(4, 29)	108	1.8	76.57	6.26
(1, 7)	162	2	96.92	9.39	(4, 45)	215	2.8	117.47	12.46
(1, 8)	148	1.8	62.31	8.57	(6, 37)	8	0.2	6.74	0.46
(1, 9)	90	1.5	52.42	5.27	(6, 38)	10	0.3	8.42	0.58
(1, 15)	243	2.8	123.84	14.08	(8, 35)	3	0.2	3.99	0.17
(1, 20)	235	3.3	57.28	13.62	(10, 36)	9	0.4	4.39	0.52
(1, 23)	174	2.5	61.69	10.08	(11, 36)	8	0.3	3.90	0.46
(1, 24)	291	3.4	77.37	16.86	(12, 42)	152	2	72.41	8.81
(1, 26)	308	3.7	76.43	17.84	(13, 42)	15	0.6	6.48	0.87
(1, 33)	403	5	98.22	23.35	(14, 15)	101	1.4	49.23	5.85
(1, 34)	248	3.3	63.74	14.37	(14, 16)	123	1.7	31.67	7.13
(1, 36)	238	3.2	137.11	13.79	(14, 36)	114	1.8	75.78	6.60
(1, 39)	262	3.3	156.74	15.18	(14, 37)	9	0.4	5.58	0.52
(1, 40)	382	4.2	169.28	22.13	(14, 38)	9	0.4	5.58	0.52
(1, 41)	333	3.8	162.32	19.29	(14, 39)	119	1.9	65.92	6.89
(1, 42)	164	2.3	83.58	9.50	(14, 40)	239	2.7	119.68	13.85
(1, 43)	171	2.3	64.41	9.91	(14, 41)	191	2.4	63.48	11.07
(1, 44)	434	4.9	211.56	25.15	(16, 39)	6	0.2	3.99	0.35
(1, 45)	445	5.4	226.78	25.78	(16, 40)	143	1.8	22.18	8.29
(2, 6)	150	1.8	76.44	8.69	(16, 41)	95	1.5	43.36	5.50
(2, 7)	151	1.7	76.95	8.75	(18, 39)	71	1.6	15.10	4.11
(2, 8)	265	6	131.52	15.35	(18, 40)	56	0.8	9.93	3.24
(2, 12)	3	0.3	0.40	0.17	(18, 41)	45	1	13.96	2.61
(2, 15)	150	2.1	66.47	8.69	(19, 40)	75	1.1	13.29	4.35
(2, 20)	377	4.5	125.30	21.84	(19, 41)	161	2.1	46.84	8.75
(2, 33)	577	6.6	145.74	33.43	(20, 33)	215	2.6	100.04	12.46
(2, 34)	422	5.2	114.07	24.45	(20, 34)	59	1	41.83	3.42
(2, 35)	265	3	131.52	15.35	(21, 33)	168	2.2	72.21	9.73
(2, 36)	237	3	119.52	13.73	(21, 34)	4	0.1	2.39	0.21
(2, 37)	155	1.9	78.99	8.98	(22, 33)	113	1.9	48.57	6.55
(2, 38)	140	1.8	71.35	8.11	(22, 34)	23	0.5	15.29	1.33
(2, 41)	256	2.8	113.44	14.83	(23, 43)	8	0.3	3.37	0.46
(3, 6)	439	4.8	184.81	25.43	(23, 44)	266	3.1	126.13	15.41
(3, 12)	376	4.4	159.96	21.78	(23, 45)	278	4.2	123.19	16.11
(3, 24)	23	0.6	17.84	1.33	(26, 44)	137	1.6	77.71	7.94
(3, 25)	10	0.3	7.76	0.58	(26, 45)	149	2.2	81.41	8.63
(3, 26)	27	0.7	19.14	1.56	(27, 44)	140	1.7	79.41	8.11
(3, 28)	17	0.4	16.87	0.98	(27, 45)	152	2.2	83.05	8.81
(3, 29)	42	1	32.57	2.43	(28, 44)	126	1.6	76.77	7.30
(3, 31)	144	1.9	73.38	8.34	(28, 45)	138	2.1	70.33	8.00
(3, 35)	201	2.7	172.80	11.65	(29, 44)	101	1.5	58.95	5.85
(3, 44)	142	1.8	80.55	8.23	(29, 45)	101	2.2	61.54	5.85
(3, 45)	154	2.3	84.14	8.92	(30, 31)	97	1.5	59.10	5.62
(4, 6)	412	4.4	173.45	23.87	(30, 44)	95	1.3	57.89	5.50
(4, 12)	359	4.4	152.72	20.80	(30, 45)	107	1.7	59.27	6.20
(4, 24)	59	0.8	41.83	3.42	(31, 44)	7	0.3	6.20	0.41
(4, 25)	74	1	52.47	4.29	(31, 45)	131	1.7	101.59	7.59
(4, 31)	171	2.1	96.99	9.91	(32, 44)	14	0.5	12.41	0.81
(4, 35)	204	2.8	175.38	11.82	(32, 45)	142	1.8	100.68	8.23
(4, 44)	169	1.9	145.29	9.79					

**Table B3:** Schedules and risk parameters of the rail services in the multimodal service network.

Train No.	47501	47503	47505	47507	21018	21020	21022	21024	21026	34029	34035
Origin	5	5	5	5	6	6	6	6	6	6	6
Loading start time	20.4	2.8	10.6	15.1	1.6	0.8	3.1	6	10.3	1.5	5.8
Loading cutoff time	20.9	3.8	11.5	15.8	3.1	2.3	4.6	7.5	11.8	3	7.3
Classification start time	20.7	3.5	11.3	15.6	2.6	2.8	4.5	6.5	10.8	2	6.3
Classification cutoff time	21.2	4	12.2	16.2	3.6	3.8	5.1	8	12.3	3.5	7.8
Departure time	21.4	4.3	12.6	16.5	4.1	4.3	5.6	8.5	12.8	4	8.3
Destination	6	6	6	6	8	8	8	8	8	10	10
Arrival time	22.3	5.1	13.4	17.4	18	18.4	18.7	18.8	19.5	7.5	10.8
Disassembly start time	22.5	5.3	13.6	17.7	18.5	18.9	19.2	18.3	19	7	10.3
Disassembly cutoff time	23	5.7	14.2	18.4	20	20.5	20.7	20	21	8.5	12
Unloading start time	22.8	5.5	14	18	19	19.4	19.7	19.8	20.5	8.5	11.8
Unloading cutoff time	23.5	6	14.5	19	20.5	21	21.2	21.3	22	10	13.3
Distance (unit: km)	17	17	17	17	273	273	273	273	273	115	115
Capacity (unit: ton)	706	894	994	762	1973	1354	1181	1541	1668	799	994
Population exposure (unit: 10^4^ people)	9.86	9.86	9.86	9.86	146.59	146.59	146.59	146.59	146.59	64.22	64.22
Environmental capacity (unit: 10^4^ ton)	13.67	13.67	13.67	13.67	219.45	219.45	219.45	219.45	219.45	92.44	92.44
**Train No.**	**34047**	**34055**	**34001**	**34045**	**47551**	**47555**	**47557**	**32101**	**32105**	**32109**	**32119**
Origin	6	6	6	6	6	6	6	6	6	6	6
Loading start time	9.3	11.8	16.1	8.6	17.9	5.5	11.7	16.3	17.1	17.3	2.8
Loading cutoff time	10.8	13.3	17.6	10.1	18.4	6.2	12.3	17.8	18.6	18.8	4.3
Classification start time	9.8	12.3	16.6	9.1	18.3	6	11.9	16.8	17.6	17.8	3.3
Classification cutoff time	11.3	13.8	18.1	10.6	18.6	6.5	12.4	18.3	19.1	19.3	4.8
Departure time	11.8	14.3	18.6	11.1	18.9	6.9	12.7	18.8	19.6	19.8	5.3
Destination	10	10	11	11	14	14	14	17	17	17	17
Arrival time	14.3	16.8	21	13.5	19.1	7	12.8	21.4	22.5	22.9	7.5
Disassembly start time	13.8	16.3	20.5	13	19.4	7.5	13.2	20.9	22	22.4	7
Disassembly cutoff time	15.5	18	22.3	14.7	19.9	8	13.7	22.4	23.8	24	8.7
Unloading start time	15.3	17.8	22	14.5	20	7.8	13.5	22.4	23.5	23.9	8.5
Unloading cutoff time	16.8	19.3	23.5	16	21	8.5	14	23.9	25	25.4	10
Distance (unit: km)	115	115	107	107	9	9	9	162	162	162	162
Capacity (unit: ton)	1066	1747	1062	1094	874	1080	972	1680	1181	1145	1786
Population exposure (unit: 10^4^ people)	64.22	64.22	59.75	59.75	4.25	4.25	4.25	80.03	80.03	80.03	80.03
Environmental capacity (unit: 10^4^ ton)	92.44	92.44	86.01	86.01	7.23	7.23	7.23	130.22	130.22	130.22	130.22
**Train No.**	**32123**	**30002**	**30004**	**30006**	**34005**	**34011**	**34037**	**34041**	**34049**	**34013**	**34025**
Origin	6	6	6	6	7	7	7	7	7	7	7
Loading start time	6.8	16.7	17.4	18.3	18.3	20.1	6.6	7.8	10.3	21.4	1.6
Loading cutoff time	8.3	18.2	18.9	19.8	19.8	21.6	8.1	9.3	11.8	22.9	3.1
Classification start time	7.3	17.2	17.9	18.8	18.8	20.6	7.1	8.3	10.8	21.9	2.1
Classification cutoff time	8.8	18.7	19.4	20.3	20.3	22.1	8.6	9.8	12.3	23.4	3.6
Departure time	9.3	19.2	19.9	20.8	20.8	22.6	9.1	10.3	12.8	23.9	4.1
Destination	17	12	12	12	10	10	10	10	10	11	11
Arrival time	11.9	21.5	22.2	23.1	23	0.8	11.3	12.4	15	2	6.1
Disassembly start time	11.4	21	21.7	22.6	22.5	0.3	10.8	11.9	14.5	1.5	5.6
Disassembly cutoff time	13	22.8	23.5	24.3	24.6	2	12.5	13.5	16.7	3.3	7.3
Unloading start time	12.9	22.5	23.2	24.1	24	1.8	12.3	13.4	16	3	7.1
Unloading cutoff time	14.4	24	24.7	25.6	25.5	3.3	13.8	14.9	17.5	4.5	8.6
Distance (unit: km)	162	121	121	121	107	107	107	107	107	99	99
Capacity (unit: ton)	1001	1505	1109	1508	1987	828	1134	1727	1836	940	1620
Population exposure (unit: 10^4^ people)	80.03	53.69	53.69	53.69	59.75	59.75	59.75	59.75	59.75	55.28	55.28
Environmental capacity (unit: 10^4^ ton)	130.22	97.26	97.26	97.26	86.01	86.01	86.01	86.01	86.01	79.58	79.58
**Train No.**	**34031**	**33912**	**33914**	**21001**	**21017**	**21019**	**21023**	**21025**	**21027**	**33039**	**33041**
Origin	7	7	7	8	8	8	8	8	8	8	8
Loading start time	3.6	20.4	0.4	19.1	2.5	3.5	4.1	6.8	11.1	18.5	20.5
Loading cutoff time	5.1	21.9	1.1	20.6	4	5	5.6	8.3	12.6	20	22
Classification start time	4.1	20.9	0.1	19.6	3	4	4.6	7.3	11.6	19	21
Classification cutoff time	5.6	22.4	1.6	21.1	4.5	5.5	6.1	8.8	13.1	20.5	22.5
Departure time	6.1	22.9	2.1	21.6	5	6	6.6	9.3	13.6	21	23
Destination	11	13	13	6	6	6	6	6	6	20	20
Arrival time	8.2	3.3	5.2	5.7	11.3	13.5	15.3	20.5	0.7	22.9	0.9
Disassembly start time	7.7	2.8	4.7	5.2	10.8	13	14.8	20	0.2	22.4	0.4
Disassembly cutoff time	9.5	4.6	6.3	7	12.7	14.5	16.6	21.6	2	23.9	2
Unloading start time	9.2	4.3	6.2	6.7	12.3	14.5	16.3	21.5	1.7	23.9	1.9
Unloading cutoff time	10.7	5.8	7.7	8.2	13.8	16	17.8	23	3.2	25.4	3.4
Distance (unit: km)	99	185	185	273	273	273	273	273	273	121	121
Capacity (unit: ton)	1469	1174	1253	1840	1990	1146	1075	1393	1495	1170	922
Population exposure (unit: 10^4^ people)	55.28	75.49	75.49	146.59	146.59	146.59	146.59	146.59	146.59	33.26	33.26
Environmental capacity (unit: 10^4^ ton)	79.58	148.71	148.71	219.45	219.45	219.45	219.45	219.45	219.45	97.26	97.26
**Train No.**	**33057**	**33061**	**33067**	**35547**	**35553**	**35557**	**35505**	**38001**	**38011**	**38027**	**40103**
Origin	8	8	8	8	8	8	8	8	8	8	9
Loading start time	8	10.1	14.5	12.7	13.6	14.9	18.6	16.3	1	13.7	15.9
Loading cutoff time	9.5	11.6	16	14.2	15.1	16.4	20.1	17.8	2.5	15.2	16.4
Classification start time	8.5	10.6	15	13.2	14.1	15.4	19.1	16.8	1.5	14.2	16.1
Classification cutoff time	10	12.1	16.5	14.7	15.6	16.9	20.6	18.3	3	15.7	16.9
Departure time	10.5	12.6	17	15.2	16.1	17.4	21.1	18.8	3.5	16.2	17.3
Destination	20	20	21	23	23	23	23	26	26	26	12
Arrival time	12.4	14.4	20	16.8	18.6	19.2	23.7	21.3	6	21	20.6
Disassembly start time	11.9	13.9	19.5	16.3	18.1	18.7	23.2	20.8	5.5	20.5	21
Disassembly cutoff time	13.5	15.7	21.2	18	20	20.5	24.7	22.5	7	22.3	21.6
Unloading start time	13.4	15.4	21	17.8	19.6	20.2	24.7	22.3	7	22	21.4
Unloading cutoff time	14.9	16.9	22.5	19.3	21.1	21.7	26.2	23.8	8.5	23.5	22
Distance (unit: km)	121	121	171	113	113	113	113	262	262	262	127
Capacity (unit: ton)	1546	994	1102	944	1239	1462	1009	856	1484	1135	864
Population exposure (unit: 10^4^ people)	33.26	33.26	49.21	82.52	82.52	82.52	82.52	225.09	225.09	225.09	94.54
Environmental capacity (unit: 10^4^ ton)	97.26	97.26	137.46	90.83	90.83	90.83	90.83	210.61	210.61	210.61	102.09
**Train No.**	**30001**	**30003**	**30005**	**40081**	**47405**	**47406**	**39101**	**39109**	**39111**	**39113**	**39115**
Origin	12	12	12	15	16	17	17	17	17	17	17
Loading start time	23.1	5.5	7.1	17.7	22.5	14.2	17.3	5.5	8.2	11.5	14.7
Loading cutoff time	24.6	7	8.6	19.2	24	15.7	18.8	7	9.7	13	16.2
Classification start time	23.6	6	7.6	18.2	23	14.7	17.8	6	8.7	12	15.2
Classification cutoff time	25.1	7.5	9.1	19.7	24.5	16.2	19.3	7.5	10.2	13.5	16.7
Departure time	25.6	8	9.6	20.2	25	16.7	19.8	8	10.7	14	17.2
Destination	6	6	6	17	17	16	18	18	18	18	18
Arrival time	28.1	10.8	12.1	22.3	25.9	17.6	21.2	9.4	12.2	15.4	18.7
Disassembly start time	27.6	10.3	11.6	21.8	25.4	17.1	20.7	8.9	11.7	14.9	18.2
Disassembly cutoff time	29.5	12	13.7	23.5	27	18.8	22.2	10.5	13.6	16.4	20
Unloading start time	29.1	11.8	13.1	23.3	26.9	18.6	22.2	10.4	13.2	16.4	19.7
Unloading cutoff time	30.6	13.3	14.6	24.8	28.4	20.1	23.7	11.9	14.7	17.9	21.2
Distance (unit: km)	121	12	12	67	52	52	44	44	44	44	44
Capacity (unit: ton)	1901	884	1392	857	1893	1107	2011	1892	1469	1242	1058
Population exposure (unit: 10^4^ people)	55.41	5.49	5.49	31.66	20.10	20.10	11.34	20.79	20.79	20.79	20.79
Environmental capacity (unit: 10^4^ ton)	97.26	9.65	9.65	53.86	41.80	41.80	35.37	35.37	35.37	35.37	35.37
**Train No.**	**39181**	**39183**	**39185**	**33201**	**33203**	**46721**	**46723**	**82114/3**	**86662/1**	**46915**	**46947**
Origin	17	17	17	20	20	24	24	24	24	25	25
Loading start time	22.7	1.5	13.7	18.3	1.9	22.5	4.8	2.5	7.9	8.6	5
Loading cutoff time	24.2	3	15.2	19.8	3.4	23	5.4	4	9.4	9.2	5.7
Classification start time	23.2	2	14.2	18.8	2.4	23	5.2	3	8.4	8.9	5.5
Classification cutoff time	24.7	3.5	15.7	20.3	3.9	23.5	6	4.5	9.9	9.6	6.2
Departure time	25.2	4	16.2	20.8	4.4	23.8	6.4	5	10.4	10	6.5
Destination	19	19	19	22	22	28	28	29	30	26	26
Arrival time	34.8	14.7	0.7	22.2	5.8	1.4	7	7	12.8	10.5	7
Disassembly start time	34.3	14.2	0.2	21.7	5.3	1.8	7.5	6.5	12.3	10.8	7.2
Disassembly cutoff time	36	15.9	1.9	23.4	7	2.8	8.1	8.2	14	11.5	7.8
Unloading start time	35.8	15.7	1.7	23.2	6.8	2.1	7.8	8	13.8	11.4	7.5
Unloading cutoff time	37.3	17.2	3.2	24.7	8.3	3	8.5	9.5	15.3	12	8.1
Distance (unit: km)	203	203	203	75	75	26	26	49	69	13	13
Capacity (unit: ton)	722	1284	1675	1156	1786	1023	1111	1987	1509	1160	1283
Population exposure (unit: 10^4^ people)	52.32	52.32	52.32	45.10	45.10	18.99	18.99	35.78	35.57	9.49	9.49
Environmental capacity (unit: 10^4^ ton)	163.18	163.18	163.18	60.29	60.29	20.90	20.90	39.39	55.47	10.45	10.45
**Train No.**	**46951**	**46944**	**46821**	**46829**	**46833**	**46835**	**46839**	**43093**	**46981**	**46983**	**46981**
Origin	25	26	28	28	28	28	28	29	31	31	31
Loading start time	21	1.2	18.5	3.9	7.5	8.6	11	10.9	20	7.5	19.5
Loading cutoff time	21.7	2	19.2	4.5	8.3	9.4	12	11.6	21	8.6	21
Classification start time	21.9	1.8	19	4.2	8	9	11.5	11.2	20.8	8.4	20.7
Classification cutoff time	22.3	2.4	19.5	5	8.8	10	12.4	12	21.7	9.2	21.6
Departure time	22.5	2.7	19.7	5.4	8.1	10.3	12.6	12.6	22	9.6	22
Destination	26	27	30	30	30	30	30	31	32	32	32
Arrival time	23	3.1	20.4	6.2	8.9	11	13.3	14	22.7	12.4	22.7
Disassembly start time	23.3	3.5	21	6.5	9.1	11.4	13.7	14.5	30	12.8	23
Disassembly cutoff time	23.9	4.1	21.5	7.1	9.8	12.3	14.4	15.2	30.8	14	23.8
Unloading start time	23.7	3.9	21.4	6.7	9.5	11.7	14	14.8	30.4	13.4	23.4
Unloading cutoff time	24.2	4.6	22.2	7.5	10.4	12.7	15	15.8	31.4	15	24.2
Distance (unit: km)	13	8	53	53	53	53	53	88	10	10	10
Capacity (unit: ton)	879	1930	1036	2143	778	1456	1220	1696	1987	972	762
Population exposure (unit: 10^4^ people)	9.49	5.84	27.32	27.32	27.32	27.32	27.32	45.36	4.94	4.94	4.94
Environmental capacity (unit: 10^4^ ton)	10.45	6.43	42.60	42.60	42.60	42.60	42.60	70.74	8.04	8.04	8.04
