# Correction: Sun, Y.; et al. Bi-Objective Modelling for Hazardous Materials Road–Rail Multimodal Routing Problem with Railway Schedule-Based Space–Time Constraints. *Int. J. Environ. Res. Public Health* 2016, *13*, 762

**DOI:** 10.3390/ijerph13100984

**Published:** 2016-09-30

**Authors:** Yan Sun, Maoxiang Lang, Danzhu Wang

**Affiliations:** 1 School of Traffic and Transportation, Beijing Jiaotong University, Beijing 100044, China; sunyanbjtu@163.com; 2 Ministry of Education Key Laboratory for Urban Transportation Complex Systems Theory and Technology, Beijing Jiaotong University, Beijing 100044, China; 3 Transportation and Economics Research Institute, China Academy of Railway Sciences, Beijing 100081, China; wangdanzhu@rails.cn

The authors wish to make the following corrections to their paper published in the *International Journal of Environmental Research and Public Health* [1].

1. In Section 3.1 *Social Risk Evaluation*, the Greek letter “π” was missed out of Equation (4), thus, the correct version of this equation should be:
  (4) $$POP_{i}=\rho_{i}\cdot \pi \cdot {\left(\sqrt[{c+d}]{\frac{Q}{\pi \cdot u\cdot a\cdot b\cdot \bar{C}}}\right)}^{2}$$
2. In Section 4.1 *Notations*, in the definition of $\left[l_{i}^{s},u_{i}^{s}\right]$, i should belong to the set N, i.e., i∈N.

The authors would like to apologize for any inconvenience caused to the readers by these changes. The changes do not affect the scientific results. The manuscript will be updated and the original will remain available on the article webpage.
